# Evaluating the Impact of Phosphorylation on the Dynamics of the Tau Protein Proline-Rich Region

**DOI:** 10.1021/acs.jctc.5c02011

**Published:** 2026-03-04

**Authors:** Johannes Stöckelmaier, Giovanni Polato, Jozef Hritz, Chris Oostenbrink

**Affiliations:** † Institute of Molecular Modeling and Simulation (MMS), BOKU University, Muthgasse 18, 1190 Vienna, Austria; ‡ Central European Institute of Technology, Masaryk University, Kamenice 5, 625 00 Brno, Czech Republic; § National Centre for Biomolecular Research, Faculty of Science, Masaryk University, Kamenice 5, 625 00 Brno, Czech Republic; ∥ Department of Chemistry, Faculty of Science, Masaryk University, Kamenice 5, 625 00 Brno, Czech Republic; ⊥ Christian Doppler Laboratory Molecular Informatics in the Biosciences, BOKU University, Muthgasse 18, 1190 Vienna, Austria

## Abstract

The proline-rich region of the tubulin-associated unit (TAU) protein is of substantial interest in understanding neurodegenerative diseases due to its interaction with bridging integrator 1 (BIN1). The associated gene *BIN1* is substantially associated with the development of Alzheimer’s disease. Previous studies have underlined the importance of the conformation of the proline-rich region of TAU and the effect of its phosphorylation. In this study, we investigate the change in compactness between a four times phosphorylated TAU fragment (210–240) compared to the unphosphorylated (non-P) form using computational means. We apply our Ensemble Reconstruction from Fragments (ERF) approach to create two unbiased conformational ensembles from which a reweighted ensemble is derived that agrees with observables from nuclear magnetic resonance experiments. The resulting shift of the radius of gyration indicates a preference for relatively compact conformations for the non-P form, while the restraints derived from the experimental data do not substantially influence the compactness of the phosphorylated peptide.

## Graphical abstract

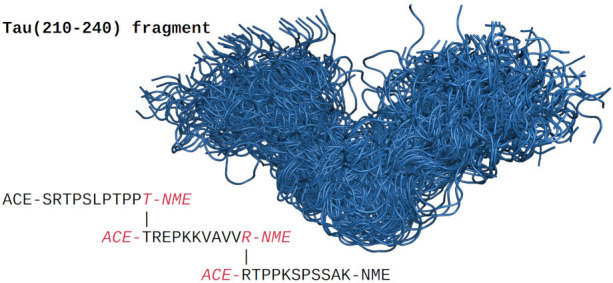

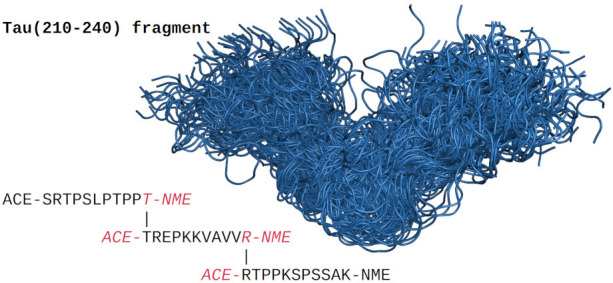

## 1 Introduction

The last decades have shown an ever-increasing understanding of biochemical processes, which have helped to improve medical treatment. While the increase in knowledge was substantial, there are still many diseases that are not yet fully understood and challenge the prospect of a healthy life until a high age. Prominent examples are Parkinson’s (PD) and Alzheimer’s disease (AD), which are the targets of intense research. It has been discovered that the tubular associated unit (TAU) protein^1^ plays a considerable role in both diseases,^2–6^ with AD being the most prevalent tauopathy. TAU is part of a group of microtubule-associated proteins and is intrinsically disordered with only a small share of secondary structure.^2,7,8^ Liquid–liquid phase separation of full-length TAU as initiation of aggregation, at least in vitro, has been reported in literature.^2,9^ In vivo, TAU is subject of several post-translational modifications,^10^ where phosphorylation has been identified to play a substantial role in the pathology of neurodegenerative diseases as increased phosphorylation levels of TAU protein have been observed as AD advances.^11–14^

Previous studies of Alzheimer’s disease have identified variants of the *BIN1* gene to substantially increase the risk of AD.^15,16^ In 2013, Chapuis et al.^17^ proposed that the *BIN1* gene mediates the AD risk by modulating the TAU pathology. The bridging integrator 1 (BIN1) protein contains a C-terminal sarcoma homology 3 (SH3) domain (BIN1 SH3).^18^ In 2015, Sottejeau et al.^19^ reported that BIN1 binds directly to the proline-rich domain of TAU. The interaction motif was located between amino acid residues 210–240, and it was shown that the interaction is weakened with increasing phosphorylation of the TAU proline-rich domain.^14,19^ Studying only a fragment of the full-length TAU is advantageous both in experiments and in computer simulations, even though one can never be entirely sure that the behavior of the fragment is relevant in the context of a longer protein, with potential long-range effects. The secondary propensities as determined for the proline-rich region of TAU (210–240)^14^ and for the full-length TAU^20^ suggest that the fragment is similarly (un)­structured in isolation as in the context of the full-length TAU.

This proline-rich region of TAU (210–240) can be described as intrinsically disordered protein (IDP), which persists in the complex with BIN1.^14^ This makes the elucidation of the structural background of the BIN1-TAU interaction difficult. IDPs lack a sustained 3D structure but are nevertheless often critical in fulfilling specific physiological roles.^21,22^ An appropriate representation requires a conformational ensemble^23–25^ capturing the structural diversity of the IDP. The nature of IDPs, which in most cases must be described using complex, multifunneled potential energy surfaces with shallow minima,^26,27^ makes them difficult to either measure experimentally or simulate computationally. From a computational standpoint, the major challenge is to sample the whole conformational space, which is in the case of IDPs very wide and thus extremely costly to calculate. Most classical approaches undersample the theoretically available conformational space and, in consequence, create a conformational ensemble, which is missing out conformations that may be relevant.

In this study, we use extensive classical molecular dynamics (MD) simulations to study the effect of phosphorylation of TAU (210–240) on the general compactness of the polypeptide. We then introduce our custom-designed Ensemble Reconstruction from Fragments (ERF) protocol, which allows us to take advantage of the weak long-range intramolecular interactions of IDPs. We use our recently developed Umbrella Refinement of Ensembles (URE) method^28^ to then reconstruct a wide ensemble of TAU (210–240), which is in agreement with the available experimental observables published by Lasorsa et al.^14,29^ Finally, we compare both sets of conformational ensembles and discuss the differences and opportunities for future research.

## 2 Methods

### 2.1 Simulation of the Full TAU Proline-Rich Region

Molecular dynamics simulations were performed for the entire region of interest (residues 210–240, SRTPSLPTPPTREPKKVAVVRTPPKSPSSAK) using the OpenMM software.^30^ The topologies for both the unphosphorylated (non-P) and phosphorylated (4P) form of the polypeptide were set up using ambertools tleap.^31^ The 4P peptide differs from the unmodified non-P peptide such that residues THR212, THR217, THR231, and SER235 were phosphorylated with singly protonated, covalently bound phosphate groups. As force field, Amber ff14SB^32^ with its extension for phosphorylated residues was chosen as well as the TIP3P^33^ water model. As the major focus of the simulations is on the effect of phosphorylation, we chose the combination of the Amber ff14SB force field and TIP3P water model for their support^34^ of phosphorylated amino acids. The advantage of having an established peptide and water force field combination—also in regard to phosphorylated groups—therefore outweighed the advantages of specific IDP focused force fields.^35,36^ As initial structures, we started with two randomly selected conformations from our previous work^37^ and added the respective end groups and post-translational modifications manually using PyMOL. To obtain comparable starting structures for both the non-P and the 4P simulations, individual preparatory simulations were performed with a small distance restraint to ensure stretched conformations, which have no memory of the initial geometry. The ω-dihedrals of the backbone were confirmed to remain in trans conformation (data not shown), and the similarity of the two forms was then checked to confirm the validity of the setup. We emphasize that these are stretched conformations, to ensure that the simulation box is large enough and that the chances of interactions with periodic copies of the peptide are minimal.

Using these stretched conformations, three production simulations for both variants were prepared using the same initial structure but different random number seeds to generate initial velocities, generating independent starting conditions. The box size was set up to have a 10 Å gap between the stretched starting conformation of the peptide and the edge of the cubic box. Sodium and chloride ions according to the Amber ff14SB force field were added to the explicit solvent to mimic an ionic strength of 0.154 M and to neutralize the charge of the polypeptide. The trajectory was calculated at a target temperature of 278 K in a constant pressure environment at 1 atm using the Langevin Middle Integrator^38^ with an integrator interval of 2 fs. The low temperature was selected to correspond to the temperature at which the NMR data was obtained. While this low temperature may come at the cost of slightly reduced sampling efficiency, we aimed to avoid sampling conformations that are not relevant at the temperature of the experiment. Bond lengths of all bonds involving hydrogen atoms were constrained with the tolerance set to 10^–4^. Long-range interactions were calculated using PME, with the real-space nonbonded cutoff set to 10 Å. The total length of simulation of each replica is 1000 ns, and the post processing of the trajectory has been performed using the MDAnalysis^39,40^ and MDTraj^41^ toolkits. The convergence of the simulations was checked by using the cumulative average of the radius of gyration.

Most postprocessing and trajectory analysis tools are designed and parametrized using standard amino acids and do not necessarily recognize modified amino acids and their alternative residue names correctly. This complicates our investigation on the effect of phosphorylated residues, as the phosphorylated residues are named differently and would be skipped in, e.g., the analysis of the secondary structure content. To mitigate this, we decided to mask the phosphorylated residues such that postprocessing tools accepted them as if they were unphosphorylated residues. Careful analysis indicated that this approach improved the completeness of the results compared to providing the nonmasked residues directly, which led to gaps and incomplete results in the analyses. Only NMR observables predicted for non-PTM residues were used for the reweighting (see below), as the validity of chemical shift predictions and the Karplus curve parameters for phosphorylated residues has not been confirmed.

### 2.2 Ensemble Reconstruction from Fragments (ERF)

The flexibility of IDPs poses a significant computational challenge, as an appropriate ensemble may consist of a large number of vastly different conformations. Classical molecular dynamics simulations, but also enhanced sampling methods, struggle to explore such a rich conformational landscape. To generate an ensemble that consists of a more complete set of conformations, the buildup of large biopolymers from small fragments has previously been explored by flexible-meccano (Bernadó et al.^42^) and hierarchical ensembles (Pietrek et al.^43^). For this study, we use our custom-designed Ensemble Reconstruction from Fragments (ERF) method based on the *fit_ener_traj* approach previously explored by Liu et al.^44^ Due to the nonlinear scaling of required computational resources with respect to simulated system size, it is beneficial to simulate smaller systems that allow a more complete exploration of the conformational landscape. Our ERF method exploits this behavior as larger molecules are broken down into smaller fragments, which are more easily simulated and characterized.

First, the original 31 amino acids long TAU (210–240) polypeptide was separated into three smaller fragments with 11, 11, and 12 residues each. To allow a later recombination of the fragments, an overlap of one residue is preserved during the fragmentation (Figure 1). As all of the fragments represent parts of an originally larger molecule, the N-termini were acetylated and the C-termini were N-methylated to avoid charged ends of the peptides. The simulations were set up similarly to the simulations of the full region of interest but performing only one replica per simulation.

**Figure 1:**
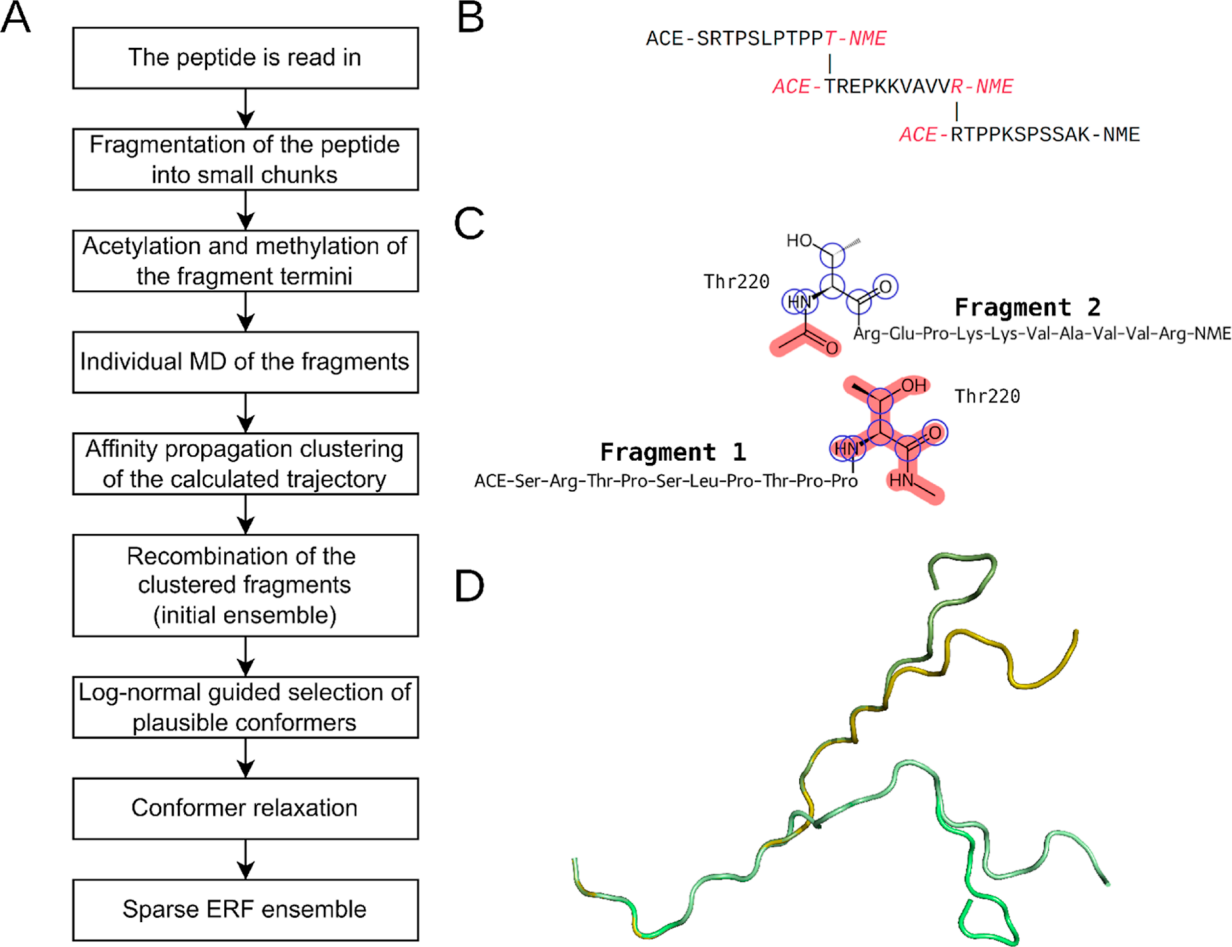
(A) The flowchart describes the main steps of the ERF protocol. A longer peptide gets fragmented into smaller parts, which are then individually simulated. The conformations obtained from these individual simulations are then clustered and reconstructed, leading ultimately to the sparse ERF ensemble. (B) The initial polypeptide TAU (210–240) was split into three fragments, each with one amino acid overlap and acetylated/N-methylated termini to avoid charged ends. When the polypeptide is reconstructed from the fragments, the overlapping amino acid is used to align the fragments. The duplicated amino acid from the overlapping region is marked in red and discarded after reconstruction. (C) The alignment and assembly of two fragments is shown in more detail. To align the fragments, neighboring fragments share one overlapping residue. The backbone, CB, and HN atoms (marked with blue circles) are used to calculate the optimal alignment. After the alignment, the now redundant end-caps and one copy of the overlapping residue (marked with red background) are removed. (D) Four conformers of the recombined ensemble are shown. They share the same conformer for fragment 1 and two different conformers for fragments 2 and 3 each. The visualization shows the branching-out, resulting from the combination of different fragment conformations together.

After finishing the MD simulation of each fragment, the water was stripped off the trajectory and the major conformations of each fragment were extracted using affinity propagation clustering^45^ with a striding of 250 ps, preference of −30, and a damping factor of 0.9. The settings were chosen to obtain around 35 conformers per fragment to allow ensemble reconstruction using an all-vs-all protocol. The non-P fragments yielded 29, 35, and 34 conformers, respectively, while the phosphorylated simulation yielded 18, 36, and 34 conformers, respectively. It is striking to note that for fragment 1, phosphorylation leads to a reduced number of distinct conformations, while this is not the case for fragment 3. Both of these fragments are doubly phosphorylated.

As a third step, a six-point atom-positional root-mean-square difference guided minimization protocol was used to recombine the clustered fragments back into the full 31 amino acid long region of interest. After the reconstruction, an initial ensemble containing all possible combinations, consisting of 34510 conformers for the non-P peptide and 22032 conformers for the 4P peptide, was obtained.

As the fourth step, each conformer of the initial ensemble was then evaluated using the Amber SB14ff force field, calculating the potential energy of each conformer. Due to the reconstruction of the region of interest, it is impossible to avoid nonphysical conformations as steric clashes occur. To avoid such conformations, an energy cutoff must be set to differentiate between plausible and implausible conformers within the initial ensemble. The profile of the potential energy distribution for a conformational ensemble obtained with this approach typically resembles the shape of a log–normal distribution. It features a long tail of conformations with high energies, making up a substantial percentage of the structures. To determine an appropriate energy cutoff, we fit the log–normal distribution onto the energy profile and defined the energy cutoff as the energy where 80% of the fitted log–normal distribution is covered (Figure S1). We therefore chose to tie the definition of the cutoff to the shape of the fitted log–normal distribution, which ensures that the thinned-out ensemble remains broad and covers most conformers with a reasonable conformation.

As the final step of the ERF protocol, each remaining conformer underwent a 20-step refinement using OpenMM and the Amber ff14SB force field to relax remaining unfavorable atom positions close to the merging points from strain as the calculation of observables is sensitive to local conformations of functional groups. The resulting final ensemble of the ERF protocol, which we call the sparse ERF ensemble, was then saved for further analysis and reweighting.

### 2.3 Reweighting

After the reconstruction of the TAU (210–240) polypeptide and potential energy-based selection, two sparse ERF ensembles consisting of 24469 (non-P) and 12109 (phosphorylated) conformers were obtained. Because interactions between residues from different fragments are largely ignored in the ERF approach, the obtained ensembles lack quantitative information about the importance (statistical weight) of each conformer. Maximum entropy-based reweighting methods^46–49^ offer the potential to obtain such quantitative information after the ensemble of interest is generated. A promising reweighting method, based on the established method of umbrella sampling, was recently introduced by us.^28^ In this approach, an initial ensemble is reweighted to a hypothetical ensemble that was generated under restraints to enforce agreement with experimental data. This tight connection of the maximum entropy formalism and umbrella sampling enables ensemble reweighting by optimizing the force constants of the hypothetical restraints that would be required to obtain a suitable ensemble, balanced with the Kullback–Leibler divergence between the native and reweighted ensembles.^28,49^ We apply the Umbrella Refinement of Ensembles method to reweight the two ensembles obtained from the reconstruction. As experimental values, ^3^ *J*-values and chemical shifts were chosen and obtained from Lasorsa et al.^14,29^ The chemical shifts were measured at 600 MHz, 278 K, and pH 7.3. Backbone assignment was performed using triple resonance solution-state NMR experiments. ^3^ *J*-values were obtained using similar conditions but at pH 6.5.

^3^ *J*-values were independently calculated using the Karplus equation^50,51^ with three different parameter sets.^52–54^ Using the three predictions, an average and a standard deviation of each coupling was calculated. The average ^3^ *J*-values were used to reweight the ensemble, while the doubled standard deviation was assumed to approximate the summed error from both simulation and experiment.

Chemical shifts were calculated for both ensembles using the software UCBShiftX of Li et al.^55^ The summed error of experiment and simulation was assumed to be twice the estimated error of the simulation, which is estimated as in the original publication of UCBShiftX.^55^

The choice of which observables to use is of substantial importance to calculate representative conformational ensembles. In Stöckelmaier and Oostenbrink,^37^ we investigated the sensitivity of predicted chemical shifts with respect to the conformation of the non-P form of TAU (210–240). With the results of our previous work in mind, a subset of chemical shifts, expected to be especially sensitive to TAU (210–240) being either completely stretched or globular, was selected. This set of chemical shifts consists of 34 chemical shifts for the non-P ensemble and 28 chemical shifts for the phosphorylated one. To assess the influence of observable choice on the result of the reweighting, an additional, more neutrally selected, subset of chemical shifts was constructed using all chemical shifts obtained from C_α_ and C_β_ atoms. The choice of these atoms was motivated by the sensitivity of secondary chemical shifts,^56–58^ a metric directly derived from C_α_ and C_β_ chemical shifts, in regard to the stable protein secondary structure. This second set of chemical shifts consists of 63 chemical shifts for the non-P ensemble and 53 chemical shifts for the phosphorylated one. Reweighting results obtained from this set of chemical shifts in combination with the ^3^ *J*-values can be found together, with a short discussion and interpretation of the results in the Supporting Information. In order to maintain the clarity and readability of this work, all data and discussion shown in the main document refer to the results of the reweighting using chemical shifts selected by Stöckelmaier and Oostenbrink.

The strength of reweighting strikes a balance between adhering to the statistical weights in the initial ensemble and enforcing agreement with the experimental data. It is described by the parameter θ. The ensemble preservation metric^28^ was used to determine a suitable value for θ to avoid overfitting. The ensemble preservation metric is a simple approach to quantifying how much of an original ensemble is maintained. Considering the native and reweighted weights of all conformations, it is estimated in the following way: weights that are increased in the reweighted ensemble contribute a value of 100% to the ensemble preservation, while weights that are lowered in the reweighted ensemble contribute the fraction of the original weight that remains. The average overall conformations determines the ensemble preservation, with a value of 100% corresponding to two identical ensembles and lower values indicating a larger divergence from the original ensemble. To avoid overfitting, a value of θ is selected, in which a considerable amount of conformations still contribute to the ensemble, indicated by a reasonable ensemble preservation. We used θ = 0.1 for refinement of the sparse ERF ensembles for both the non-P and the 4P peptides. See Stöckelmaier et al.^28^ for more details on this approach.

To compare the compliance of the simulated observables with the experiment, the root-mean-square deviation (RMSD) between the experimental ^3^ *J*-values and chemical shifts were calculated. The impact of the phosphorylation on the compactness of TAU (210–240) in an aqueous environment was further analyzed by using the radius of gyration (RGYR) of the peptide. Dictionary of Secondary Structure of Protein analysis (DSSP) as implemented in MDAnalysis was performed to estimate occurrences of secondary structure of the backbone and differences between the peptides. The trajectories of the three classical simulations of the full region of interest TAU (210–240) were merged ahead of analyzing the secondary structure, whereas the sparse ERF ensemble was analyzed both before and after the reweighting.

## 3 Results

### 3.1 Agreement with Experimental Data

The two classical MD simulations with three replicas each and the two sparse ensembles obtained by the ERF method were used to calculate the observables, as described in the previous chapter. These predicted observables were compared to the experimental data obtained from Lasorsa et al.^14,29^ The comparison gives insights into the ability of each method to reproduce the observed data using ensemble averages of the predicted data. Figure 2 shows the root-mean-square deviation (RMSD) for chemical shifts and ^3^ *J*-values for the MD-replicas as well as for the sparse ERF ensembles both before and after the reweighting using ^3^ *J*-values and the chemical shifts selected from our previous work. The deviation for the individual observables before and after reweighting is shown in Section S7 of the Supporting Information.

**Figure 2:**
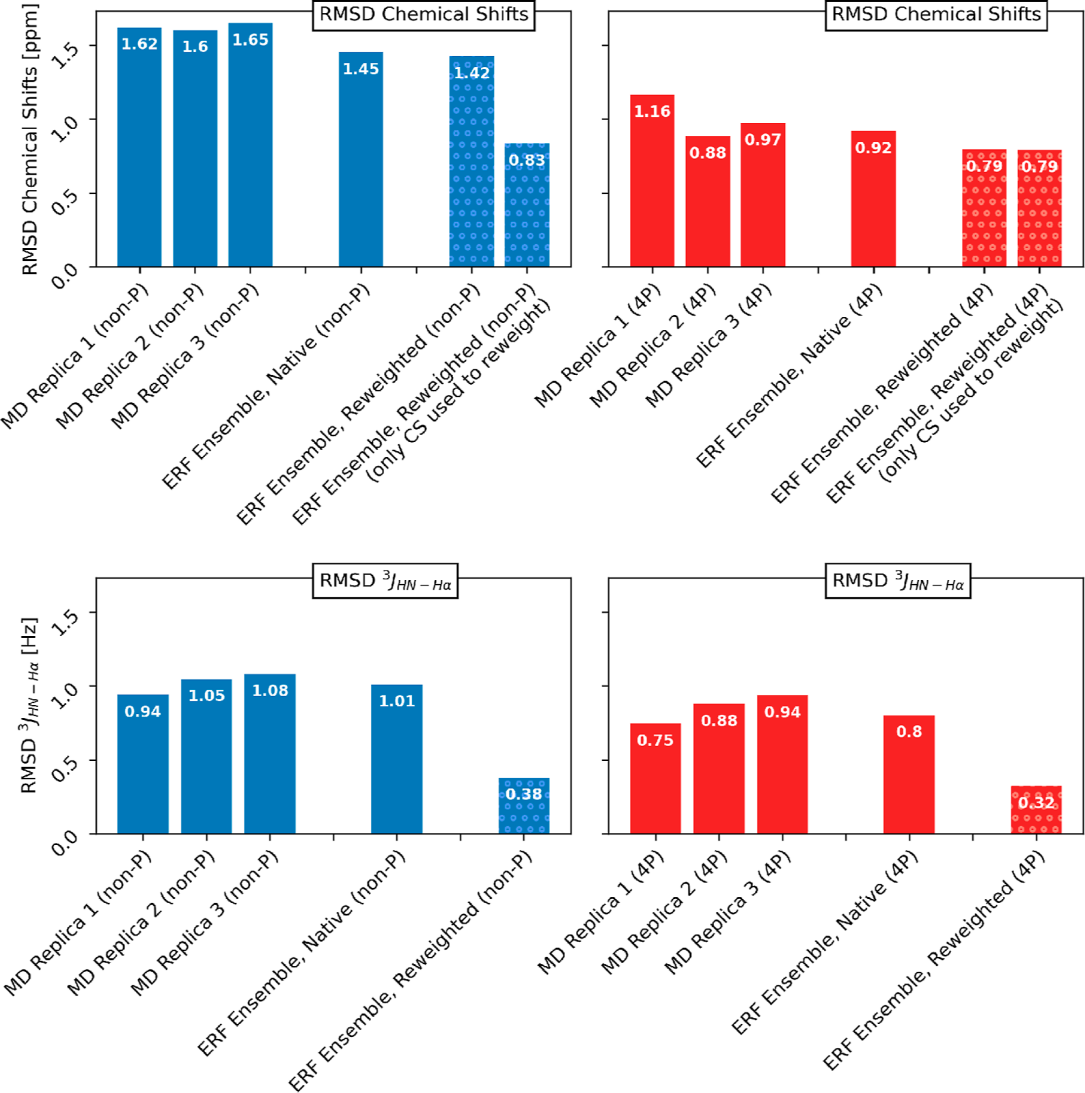
Root-mean-square deviation (RMSD) between the experimental data and simulated ensemble-averaged observables from the MD trajectories and the sparse ERF ensemble before (plain bars) and after (dotted bars) the reweighting, using ^3^ *J*-values and the chemical shifts identified in Stöckelmaier and Oostenbrink^37^ The reweighting strength was set to θ = 0.1. Blue bars show results from the non-P peptide, whereas red bars show results from the phosphorylated peptide. The native ERF ensemble shows the RMSD before the reweighting considering all chemical shifts. The reweighted ERF ensemble was evaluated using all chemical shifts and only the chemical shifts used for the reweighting.

The three MD simulations of the non-P peptide show relatively similar RMSD-values for both ^3^ *J*-values and chemical shifts, while the variability of the RMSD is larger with the 4P-peptide. Even though the variability in the RMSD is slightly higher, the observables calculated from the simulations of the phosphorylated peptide are in better agreement with the experimental data.

The RMSD of the native (before reweighting), sparse ERF ensemble is similar to or even smaller in value compared to the classical MD-simulations, an observation that can be made for both ^3^ *J*-values and chemical shifts. Interestingly, both the RMSD of the ^3^ *J*-values and the chemical shifts for the 4P peptide are again lower than the RMSDs of the non-P peptide.

Reweighting of the sparse ERF ensemble using all ^3^ *J*-values and the selected chemical shifts show a substantial improvement of the RMSD for ^3^ *J*-values and for the chemical shifts used in the reweighting. The RMSD calculated using all available 158 (non-P) and 134 (4P) chemical shifts did not show a substantial change for the non-P ensemble, while the RMSD of the 4P peptide improved from 0.92 to 0.79 ppm, the same value as the RMSD calculated using only the selected chemical shifts used in the reweighting.

### 3.2 Analysis of Compactness

The RGYR was chosen as a metric to describe the compactness of the polypeptide. It allows us to follow complex conformational changes with a single scalar to track the dynamics and preferred compactness of the polypeptide.

The MD-simulations started from a deliberately stretched initial conformation and showed a significant compaction of TAU (210–240) within the first 200 ns for both the non-P and 4P forms of the peptide (Figure 3). This initial collapse occurred faster in the non-P simulation, leading to a slightly more compact and consistent predicted RGYR between 10 and 15 Å even though occasional and temporary extensions toward 20 Å can be witnessed. The MD-simulation of the 4P peptide shows a similar compaction, with the maxima in the RGYR observed in the second half of the simulation being slightly smaller than for the non-P peptide. On the other hand, the kernel density estimates for RGYR of both sets of simulations (Figure 3C and D) show a slightly increased occurrence of larger values of RGYR for the 4P peptide.

**Figure 3:**
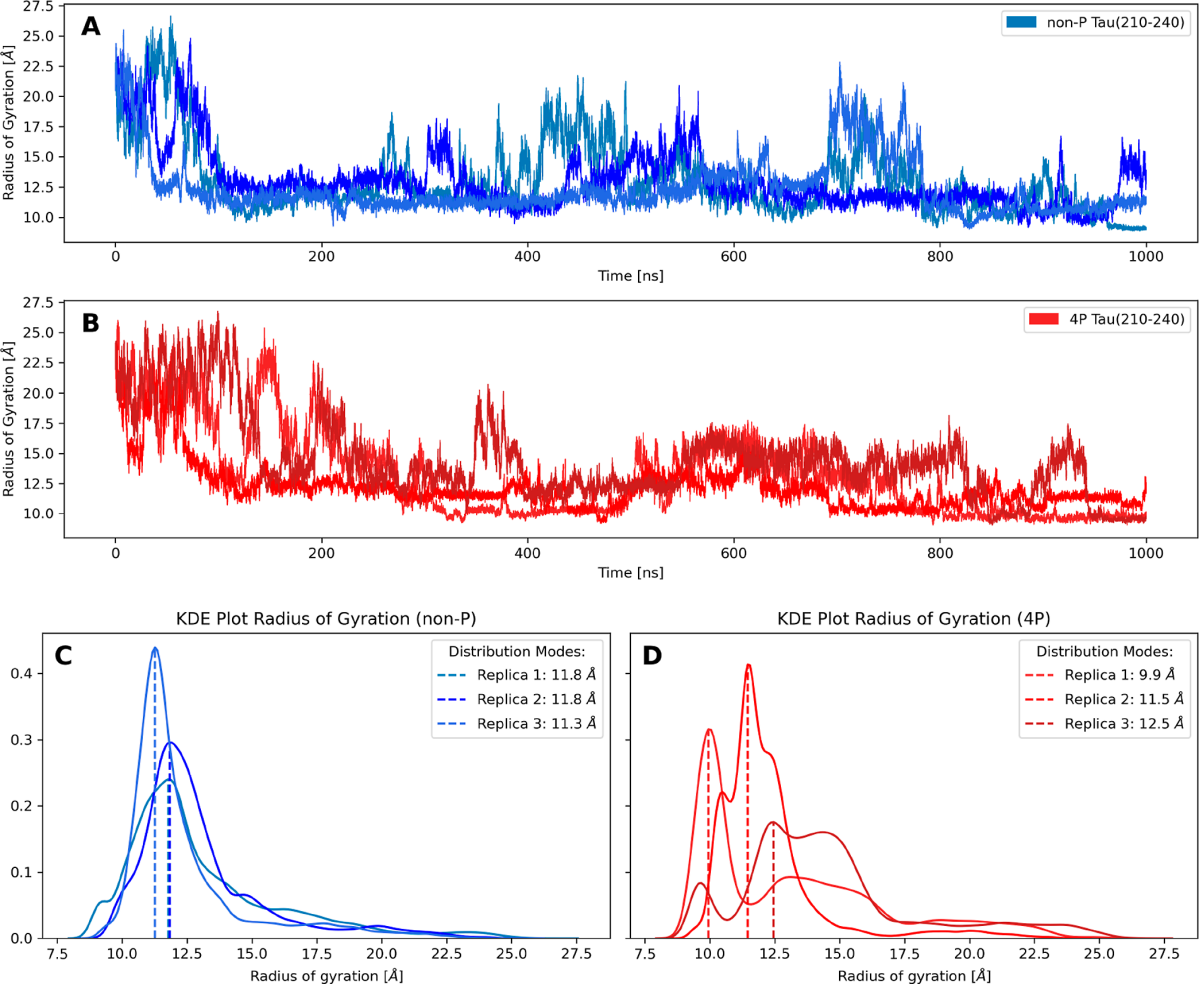
Radius of gyration (RGYR) is used to describe the compactness of the peptide. (A) RGYR during the three MD-simulations of the non-P peptide as a function of simulation time. (B) RGYR for the phosphorylated peptide as a function of simulation time. (C) Kernel density estimate (KDE) plot of the data shown in panel A. All three replicas are in agreement that the majority of the ensemble population features a RGYR between 10 and 15 Å. (D) KDE plot of the phosphorylated peptide, which indicates also an expected RGYR between 10 and 15 Å. The KDE plot of the 4P peptide shows a more diverse set of RGYR values, which may indicate an increased occurrence of slightly higher RGYR even though the modes are comparable to the non-P peptide.

The RGYR of the ensembles obtained from the ERF method shows the property to be normally distributed for both the non-P and 4P ensemble ahead of the reweighting (Figure 4, dashed lines). The distributions reach from approximately 10 Å for the most compact structures and up to 27 Å for the most stretched ones. This is consistent with our findings in ref 37 where we performed extensive simulations to find the most compact and most stretched conformation of the non-P TAU (210–240) peptide.

**Figure 4:**
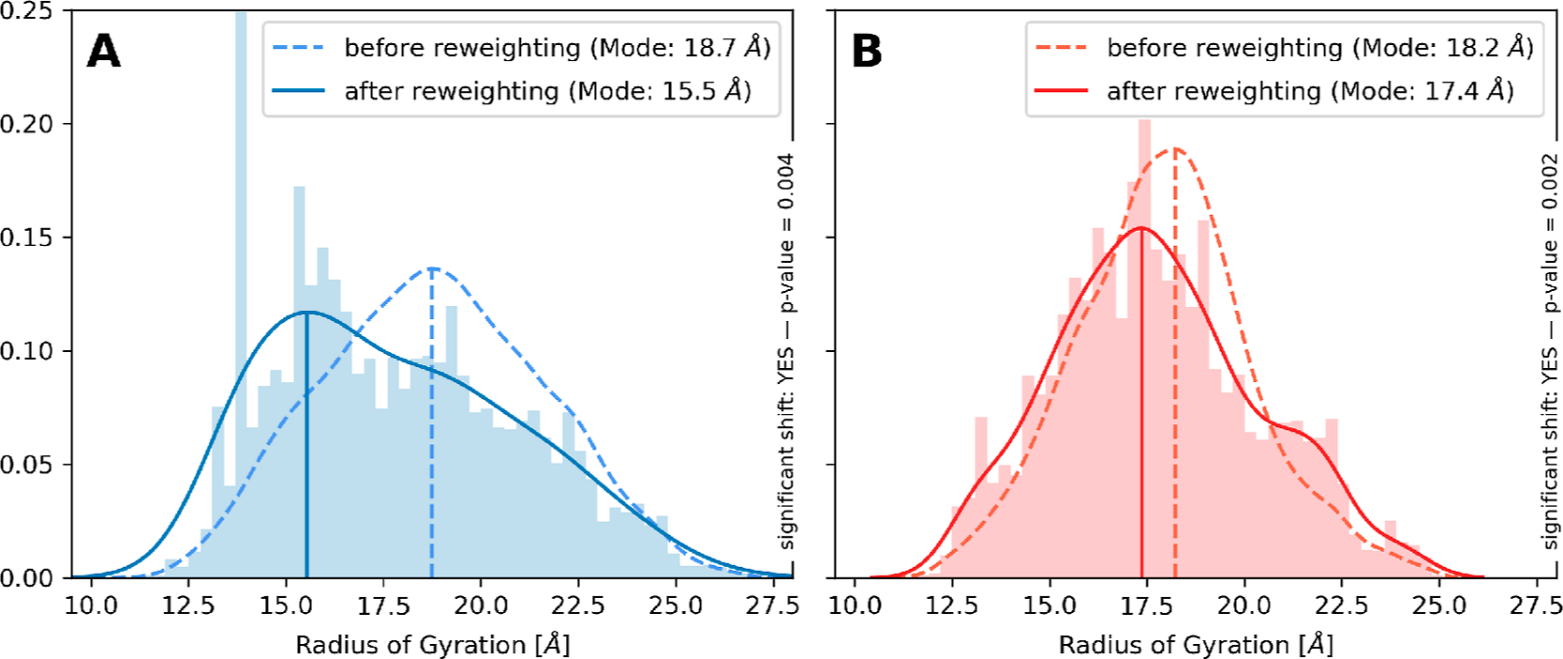
Shift of the RGYR induced due to the reweighting is shown for both the non-P (blue) and 4P peptide (red). Each plot shows the ensemble distribution of the RGYR before the reweighting (dashed line) and after the reweighting (solid line). The bars in the background represent the histogram of the reweighted ensemble. The reweighting was performed using ^3^ *J*-values and the chemical shifts recommended from Stöckelmaier and Oostenbrink^37^ The reweighting strength was set to θ = 0.1. The reweighted non-P ensemble shows a shift toward lower RGYR, indicating a preference for more compact conformations. The 4P ensemble on the other hand shows no preference for either higher or lower radii but largely maintains the initial distribution.

Reweighting of both ensembles leads to a significant shift in the mode of the population. For the non-P peptide, the mode of the kernel density shifts down from 18.7 Å (dashed line) toward 15.5 Å (solid line) after the reweighting, indicating that the experimental data points toward a significantly more compact ensemble than observed in the unbiased native ERF ensemble. The reweighted 4P ensemble shows also compaction but only a minor change from 18.2 Å down toward 17.4 Å compared to the initial ensemble. While this shift is still significant, the shift is considerably smaller for the phosphorylated peptide. The histogram in the background of Figure 4 visualizes the distribution of compactness after the reweighting, showing strongly populated bins between 12.5 and 17.5 Å for the non-P peptide, an observation that cannot be made with the reweighted 4P ensemble.

Figure 5 gives a visual representation of the conformational ensembles before (panels A and C) and after (panels B and D) reweighting. The slight compaction of the non-P peptide upon reweighting can be observed from a comparison of panels A and B, while the compaction between the non-P and 4P peptide can be seen by comparing panels B and D, but it remains a rather subtle effect.

**Figure 5:**
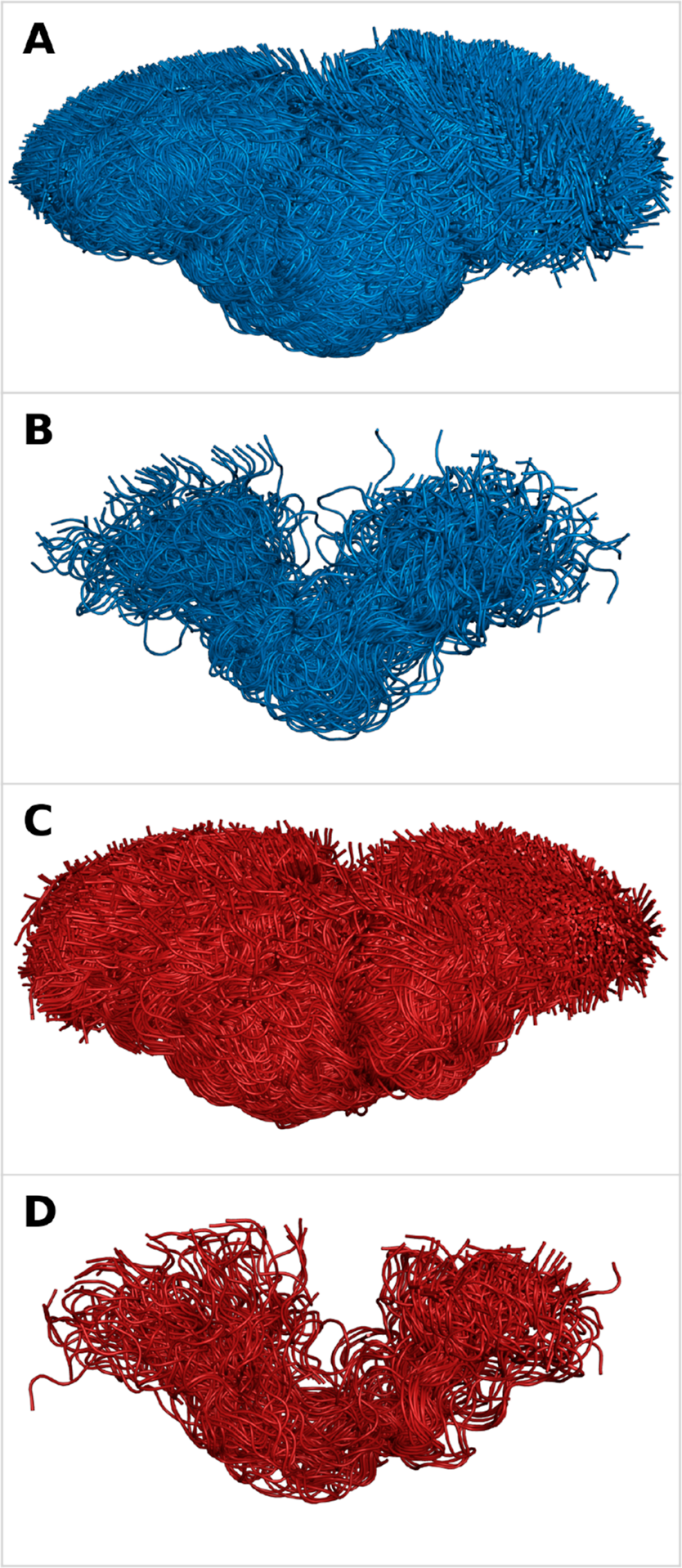
To visualize the TAU (210–240) ensembles, the full backbone of each conformer was aligned. Figure A (non-P) and C (4P) show the sparse ERF ensembles of TAU (210–240) before the reweighting. Figure B (non-P) and D (4P) show only the top 1% of the conformations with the highest statistical weights in the ERF ensemble after reweighting. Note that the non-P ensembles consist of more frames than the 4P ensembles.

### 3.3 Secondary Structure

The DSSP analysis in Figure 6 shows the observed occurrence of secondary structure elements throughout the entire polypeptide. The MD-trajectories of the non-P and 4P peptide show a small share of helically structured conformers between residues 236 and 239, which is more prominently observed in the non-P ensemble obtained from the ERF method, both before and after the reweighting. While such a secondary structure also exists in the MD-trajectory and the sparse ERF ensemble of the 4P peptide, it rather gets reduced during ensemble optimization.

**Figure 6:**
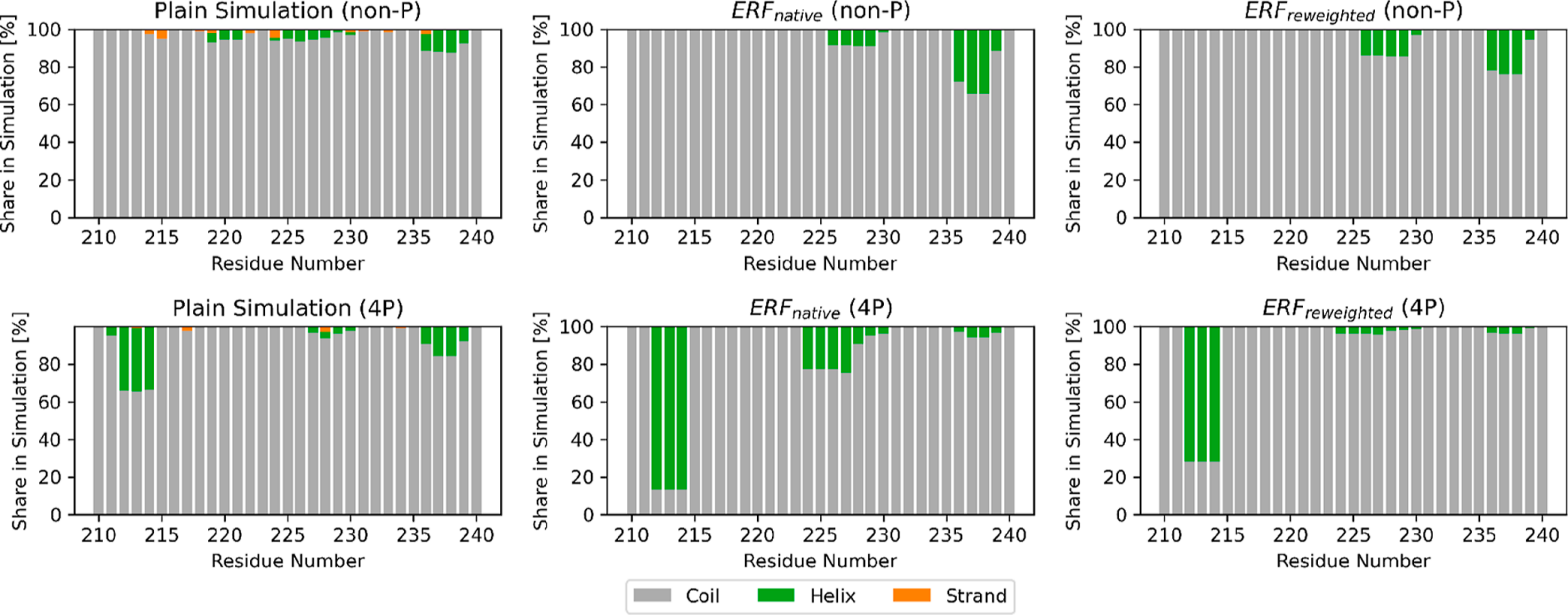
Analysis of the secondary structure of the conformers shows that the polypeptide remains in a coil-like conformation most of the time. Gray represents a coil-like secondary structure, while green suggests a helical one. Helix represents the sum of α-helix, π-helix, and 3_10_-helix; Strand represents an extended strand participating in a beta ladder (parallel and antiparallel). Figures labeled with ERF_native_ and ERF_reweighted_ refer to the sparse ERF ensemble before and after reweighting, respectively.

The MD-trajectories of the phosphorylated polypeptide suggest helix-like structures in the region of residues 212–214, which is dominant in the native ERF ensemble. This preference for a helical structure at the beginning of the peptide remains after reweighting, albeit to a slightly lesser extent.

Between residues 223 and 227, the sparse ERF ensemble of the phosphorylated polypeptide also suggests helix-like structures, but its relevance is almost removed after the reweighting. This is in agreement with the MD-trajectories, which also show very little helical structures at this position. Overall, it is clear that the great majority of the polypeptide remains in a coil-like structure, which is in agreement with our previous study of TAU (210–240).^37^

### 3.4 Charges

The distribution of charges was analyzed using a visual representation. Figure 7 visualizes the top 1% of conformations for both the non-P and 4P peptides after reweighting, sorted according to their conformational weight. Additionally, formal charges of the charged amino acids and phosphate groups are visualized at their approximate place, with negative charges colored in red and positive charges colored in blue. Big spheres indicate one formal charge, while two small spheres indicate a charge that is distributed over two atoms. The additional negative charges of the phosphorylated peptide can clearly be seen as additional red dots, but this does not lead to an obvious redistribution of the positive (blue) charges. The formal charge of the 4P peptide is plus two, while it is plus six for the non-P peptide. For the visualization, the conformations of the ensemble were RMSD-fitted onto each other. Therefore, each conformation featuring a bended shape gets fitted into the u-shaped form of the ensemble. As only statistically unlikely conformations like perfectly stretched or very globular would avoid getting fitted into this shape, the resulting ensemble appears u-shaped.

**Figure 7:**
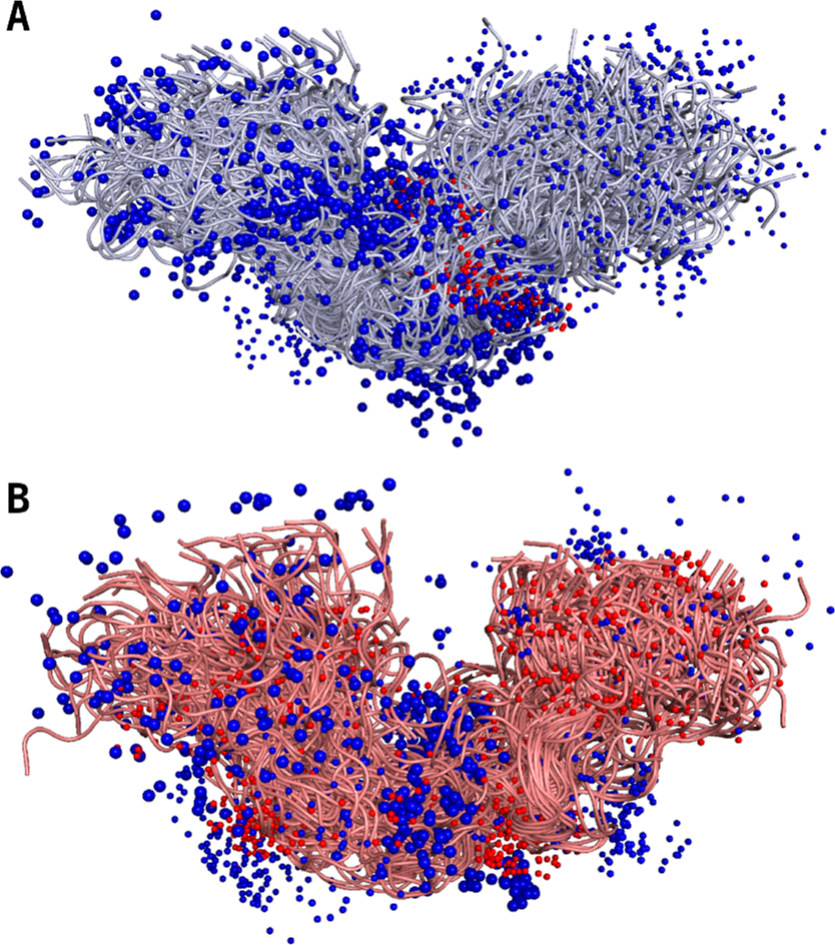
Formal charges are visualized in the top 1% ensemble of the non-P (A) and 4P (B) peptide. Formal charges of the charged amino acids and phosphate groups are visualized at their approximate place, with negative charges colored in red and positive charges colored in blue. Big spheres indicate one formal charge, while two small spheres indicate one charge that is distributed over two atoms. The formal charge of the 4P peptide is plus two, while it is plus six for the non-P peptide.

### 3.5 The Kish Effective Sample Size

The size of a conformational ensemble is typically reduced by reweighting. To estimate the number of conformations that are still effectively contributing, the effective sample size as defined by Kish^59^ can be used. Typically used in survey statistics, it indicates the size of an unbiased ensemble with a similar level of error. In the context of ensemble reweighting, we interpret the effective sample size as the number of conformers that still contribute statistically significantly to the conformational ensemble.^60^ If each conformer has the same statistical weight of 1/*N*, the effective sample size would evaluate to *N*, with a Kish ratio of 1. Accordingly, for a set of weights where only one conformer has the weight of one, the effective sample size would evaluate to a Kish ratio of 1/*N*.

The reweighting, using ^3^ *J*-values and the chemical shifts recommended from Stöckelmaier and Oostenbrink,^37^ leads to a Kish effective sample size, which is much smaller than the number of conformations in the native ERF ensemble (Table S1 in the Supporting Information). There are also substantial differences when comparing the reweighting of the non-P and 4P ERF ensembles. While the conformational ensemble of the non-P state is larger before the reweighting (24469 conformers), its effective sample size shrinks to only 158. In contrast, the unbiased ensemble of the 4P state is smaller (12109 conformers), but it retains an effective sample size of 985 after reweighting. This finding is in agreement with the assessment of the RGYR distribution in Section 3.2, which sees a larger shift of the RGYR-mode of the non-P ERF ensemble.

## 4 Discussion

The influence of phosphorylation on the conformational ensemble of the proline-rich region of the TAU protein has been investigated by using extensive computational simulations for both the phosphorylated and the non-P form. The sparse ERF ensemble carries uniform initial statistical weights for each conformer. Due to the fact that all conformations with unfavorable potential energies were disregarded, we consider each conformer of the initial ensemble as equal. The resulting ensemble offers a very diverse set of conformations, embedding only very local prior knowledge originating from the fragment simulations. The ensemble may therefore be considered as blind to previous knowledge. The distribution of the RGYR nicely follows the shape of a normal distribution, indicating a good initial coverage of the conformational space. Despite its advantages, such an ensemble is not a description of the real TAU (210–240) ensemble but a pool of structures designed to be as diverse as is attainable.

The reweighting is designed to subsequently pick a subensemble with better agreement with the experimental data but which is still as similar as possible to the nonreweighted ensemble. The resulting ensemble is then, according to the theory of maximum entropy methods, the ensemble with the least added bias that is in agreement with the experimental data provided (3*J*-couplings and chemical shifts) at a specific strength of reweighting. We do not claim that the reweighted ensemble is the definitive elucidation of the TAU (210–240) conformational ensemble but an indication of which selection of structures is beneficial to gain better agreement with the experimental data used for the reweighting. Figure 4 shows that in the case of the non-P peptide, it is necessary to disregard many conformations with a higher RGYR, creating a more compact ensemble. On the other hand, the 4P ensemble does not show such a shift, meaning that any disregarded conformations are not associated with having a specific RGYR.

The main challenge of ensemble reweighting remains the careful and unbiased selection, validation, and curation of experimental data. The quality of ensemble reweighting can be only as good as the data provided to the refinement. For this work, we decided against using all available chemical shifts to keep a balance with the ^3^ *J*-values, such that the influence from both types of observables is similar while reweighting. In the Supporting Information, we discuss results obtained using C_α_ and C_β_ chemical shifts together with the ^3^ *J*-values instead, which are similar to the results presented here.

The RMSD between simulation and experiment for both the ^3^ *J*-values and chemical shifts shows that the error of the sparse ERF ensemble before reweighting is similar in value compared to the MD-simulations, suggesting that the experimental data are more informative of local conformations than global changes. For longer range effects, other experimental observables likely need to be included. While it is clear that the reweighting mostly optimizes the selected observables used for the reweighting, also a slight improvement of the overall RMSD taking all chemical shifts into account was observed.

While the three 1 μs simulations for both the non-P and the 4P peptides are quite extensive, the number of larger conformational changes remains limited: a single collapse from the extended structure and a handful of excursions to less compact conformations is observed. This raises questions as to the true convergence of the ensemble average properties. The convergence behavior was investigated using an evaluation of the forward and backward cumulative RGYR (Supporting Information Figures S11 and S12). It was shown that for both the non-P and 4P forms, one out of three replicas seemed converged with the remaining two being close to convergence.

The phosphorylation of the polypeptide adds negative charges to the molecule, changing its formal charge from +6 (non-P) to +2 (4P). Despite the more balanced charge, we could not observe a preferred orientation of the phosphates into the center of the U-shaped peptide but rather a quite diverse orientation. The analysis of the secondary structure confirmed that the polypeptide contains only very little stable secondary structure. The vast majority of observed conformations could be classified as coil like structures, with only small parts showing the occurrence of a helical secondary structure.

From a technical perspective, the exact choice of force field is known to substantially influence the conformations seen for IDPs substantially. In Lasorsa et al.^29^ very similar simulations were performed for the same peptides, using the AMBER99sb-ildn and Charmm36m force fields with the TIP4P-D water model. Qualitatively similar time series of RGYR were obtained with one conformational collapse and a few excursions to less compact structures. From that work, the authors concluded that the phosphorylated peptide has a preference for more compact structures than the non-P peptide. In contrast, with the ERF approach, the influence of the force field is limited to local, short-range interactions between the amino-acids. Accordingly, the initial ensemble remains very wide, with a much larger range of RGYR-values. A small number of experimental data points are subsequently used to induce shifts in the conformational ensemble. In this work, we observe a slight shift toward more compact structures for the non-P peptide but not for the 4P peptide.

Note that according to Figure 2, the overall agreement of the MD-generated and the initial ERF-ensemble with the experimental data is very comparable, suggesting that the metric may hit an upper limit at which one cannot decide which one is more appropriate. The most conservative choice would be to follow the ensembles that suffer least from convergence issues or force field bias but that identify conformational shifts based on the experimental data.

While proteins with protein post-translational modifications (PTMs) are common in nature, the coverage of such modifications in simulation tools can be limited. Molecular dynamics simulation allows such PTMs to be simulated, and all parameters used in this work are part of official force field distributions. Nevertheless, it has to be taken into account that such force field additions may not reach the same level of quality as those of the standard parameters.

## 5 Conclusion

The impact of phosphorylation on the conformation of polypeptides has been a topic of discussion in the scientific literature since decades. Especially in the case of TAU protein, the conformational ensemble is of great interest to obtain better understanding of the physiological processes that may lead to Alzheimer’s disease.

In this report, we evaluated the flexibility and compactness of both the non-P and phosphorylated forms of TAU (210–240). Starting from a deliberately stretched initial conformation, rapid compaction can be observed for both forms. While the simulations indicate more rapid compaction of the non-P form, reversible extensions to more stretched conformers can also be observed. The 4P form of the TAU polypeptide shows similar compaction during the MD simulations, with a minor trend of sharing slightly more stretched conformations in its conformational ensemble. On the other hand, substantial expansions to higher RGYR observed in the non-P trajectory are much rarer in the 4P trajectories.

The ERF method allowed us to create an unbiased initial ensemble, covering the entire space of expected RGYR. The reweighting of the phosphorylated ensemble did not show any specific preference of RGYR. In contrast, the reweighting of the non-P ensemble resulted in a substantial shift of expected RGYR values toward more compact conformations.

## Supplementary Material



## Data Availability

Scripts and input files to rerun the simulations and data analysis can be downloaded from 10.5281/zenodo.17802794. Please note that within the toolchain the non-P ensemble is named *apo* and the 4P ensemble is named *phos*.

## Notes

Description of the conformer selection process of the ERF protocol, the results and analysis of reweightings using the chemical shifts identified form Stöckelmaier et al.^37^ with θ = 0.01 and θ = 0.1 as well as C_α_ and C_β_ chemical shifts with θ = 0.1, visualization of the conformer selection, plots of agreement with experimental data, analysis of compactness,r DSSP results, the Kish effective sample size, convergence analysis, and individual per-observable errors (PDF)

## #

Interdisciplinary Nanoscience Center (iNANO) and Department of Chemistry Aarhus University, Gustav Wieds Vej 14, 8000 Aarhus, Denmark
