# Supplementary material for: Evaluating the Impact of Phosphorylation on the Dynamics of the Tau Protein Proline-Rich Region
Source: J Chem Theory Comput. 2026 Mar 4;22(6):2702–13. doi: 10.1021/acs.jctc.5c02011 (PMC13019626; doi:10.1021/acs.jctc.5c02011)
Supplement: Supplementary file 1 [file ct5c02011_si_001.pdf]

Evaluating the Impact of Phosphorylation on the Dynamics of the Tau Protein Proline-Rich Region

Supporting Information

Johannes STÖCKELMAIER[1,6], Giovanni POLATO[1], Jozef HRITZ[2,3,4] and Chris OOSTENBRINK[1,6]

1. Institute of Molecular Modeling and Simulation (MMS)
BOKU University, Muthgasse 18, 1190 Vienna, Austria
2. Current address: Interdisciplinary Nanoscience Center (iNANO) and Department of Chemistry
Aarhus University, Gustav Wieds Vej 14, 8000 Aarhus, Denmark
3. Central European Institute of Technology, Masaryk University, Kamenice 5, 625 00 Brno, Czech Republic
4. National Centre for Biomolecular Research, Faculty of Science, Masaryk University, Kamenice 5, 625 00 Brno, Czech Republic
5. Department of Chemistry, Faculty of Science, Masaryk University, Kamenice 5, 625 00 Brno, Czech Republic
6. Christian Doppler Laboratory Molecular Informatics in the Biosciences
BOKU University, Muthgasse 18, 1190 Vienna, Austria

February 12, 2026

*) Corresponding author: chris.oostenbrink@boku.ac.at

1 Conformer Selection

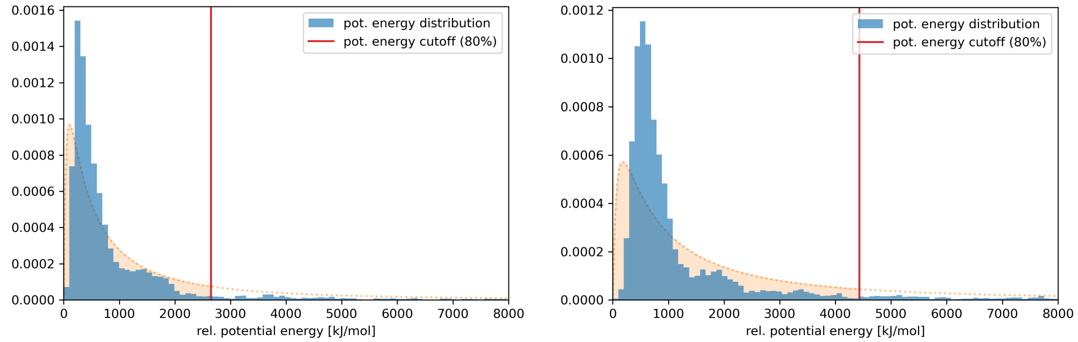

Figure S1: Due to the reconstruction of the region of interest, it is impossible to avoid non-physical conformations as steric clashes occur. To avoid such conformations, an energy cutoff has to be set to differentiate between plausible and implausible conformers within the initial ensemble. The potential energy distribution of the recombined conformations can be modeled using a log-normal distribution. For both the non-P (left figure) and the 4P (right figure) ensemble, a considerable number of conformers is close to the minimum-energy conformation. As an energy cutoff, the energy where 80% of the log-normal distribution is covered, is chosen. The selected ensemble consists of 70.9% of the initial ensemble in the non-P and 55.0% in the 4P case.

2 Agreement with Experimental Data

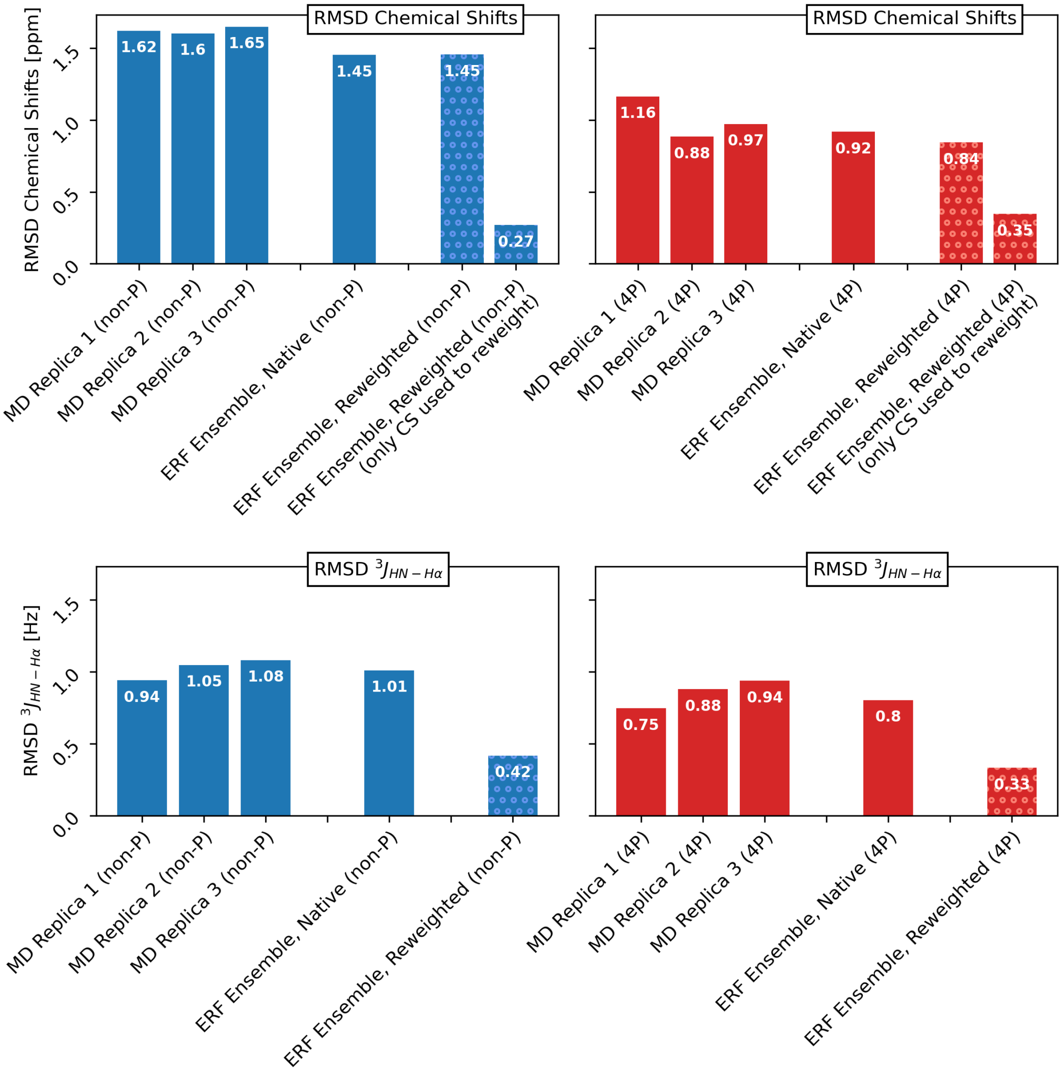

Figure S2: The root-mean-square deviation (RMSD) between the experimental data and simulated ensemble-averaged observables from the MD trajectories and the ERF ensemble before (plain bars) and after (dotted bars) the reweighting, using ^3J-values and C_α and C_β chemical shifts. The reweighting strength was set to θ = 0.1. Blue bars show results from the non-P peptide, whereas red bars show results from the phosphorylated peptide. The native ERF ensemble shows the RMSD before the reweighing considering all chemical shifts. The reweighted ERF ensemble was evaluated using all chemical shifts and only the chemical shifts used for the reweighting.

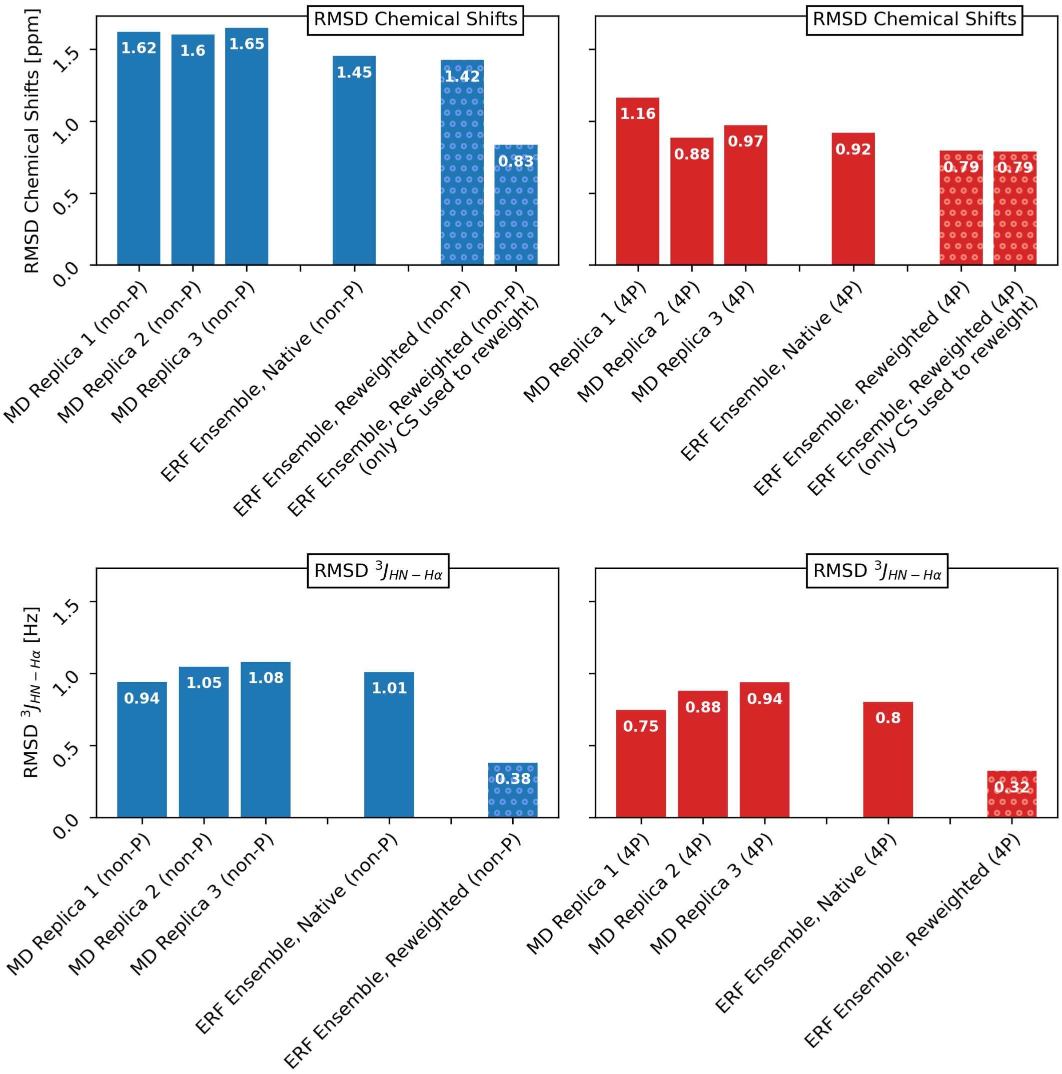

Figure S3: The root-mean-square deviation (RMSD) between the experimental data and simulated ensemble-averaged observables from the MD trajectories and the ERF ensemble before (plain bars) and after (dotted bars) the reweighting, using ^3J-values and the chemical shifts identified in Stöckelmaier et al. (PCCP 2024, 26, 23856). The reweighting strength was set to $\theta = 0.1$. Blue bars show results from the non-P peptide, whereas red bars show results from the phosphorylated peptide. The native ERF ensemble shows the RMSD before the reweighing considering all chemical shifts. The reweighted ERF-ensemble was evaluated using all chemical shifts and only the chemical shifts used for the reweighting. Repetition of figure 3 of the main manuscript, for ease of comparison.

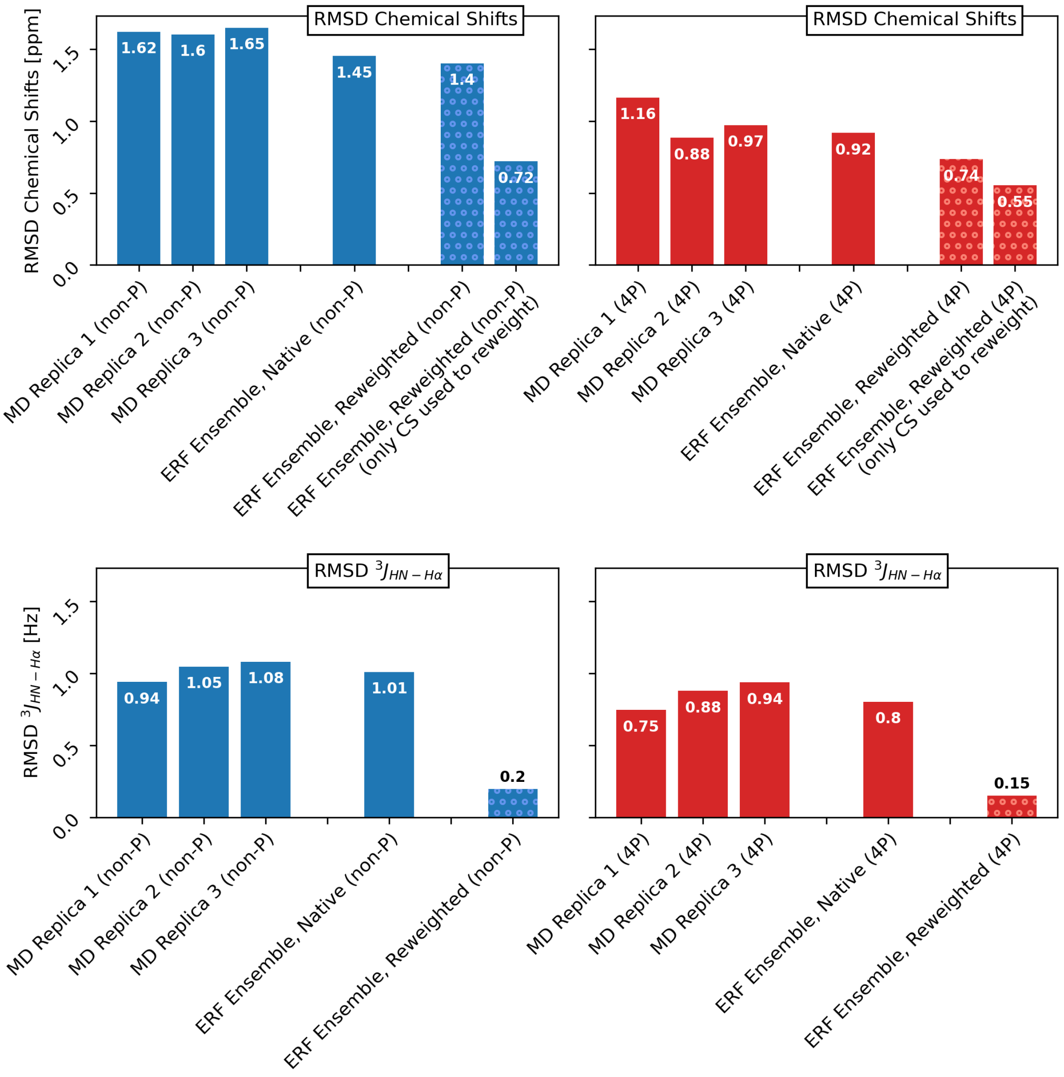

Figure S4: The root-mean-square deviation (RMSD) between the experimental data and simulated ensemble-averaged observables from the MD trajectories and the ERF ensemble before (plain bars) and after (dotted bars) the reweighting, using ^3J-values and the chemical shifts identified in Stöckelmaier et al. (PCCP 2024, 26, 23856). The reweighting strength was set to $\theta = 0.02$. Blue bars show results from the non-P peptide, whereas red bars show results from the phosphorylated peptide. The native ERF ensemble shows the RMSD before the reweighing considering all chemical shifts. The reweighted ERF-ensemble was evaluated using all chemical shifts and only the chemical shifts used for the reweighting.

3 Analysis of Compactness

Figures S5, S6 and S7 show the change in compactness of both ensembles induced by the reweighting to improve agreement with experimental data. Using the two different sub sets of chemical shifts in figure S5 and S6, a qualitatively similar shift towards more compact conformations can be observed. This shift is stronger with the chemical shift selection adapted from our previous work Stöckelmaier et. al. (PCCP 2024, 26, 23856) where conformationally

sensitive chemical shifts were intentionally selected. Figure S7 shows a reweighting result from the same set of chemical shifts but with stronger reweighting ($\theta = 0.02$ instead of $\theta = 0.1$). The histogram shows that the ensemble gets substantially more distorted with a strong bias towards a radius of gyration between 13 and 17 Å.

In our previous work, we intentionally selected chemical shifts that are predicted to differentiate between a very compact and a very stretched conformation of non-P TAU(210-240). The boosting of some conformers with a higher radius of gyration of around 22.5 Å may indicate a selection bias introduced due to this selection methodology. It may also emphasize that conformationally sensitive chemical shifts lead to substantial effects in a reweighting exercise.

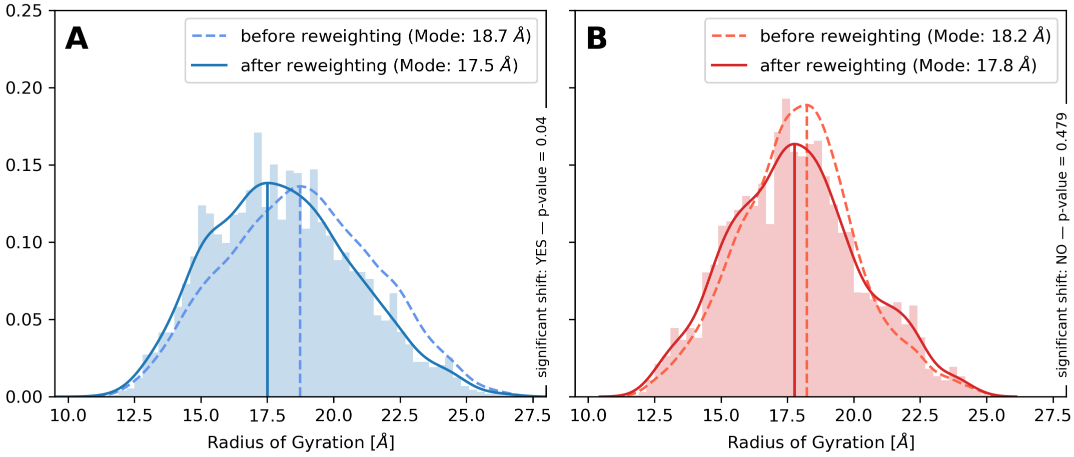

Figure S5: The kernel density estimation of the radius of gyration is shown for both the non-P (blue) and 4P peptide (red). Each plot shows the ensemble distribution of the radius of gyration before the reweighting (dashed blue line) and after the reweighting (solid line). Reweighting was based on the ^3J-values and C_α and C_β chemical shifts. The reweighting strength was set to $\theta = 0.1$. The bars in the background represent the histogram of the reweighted ensemble.

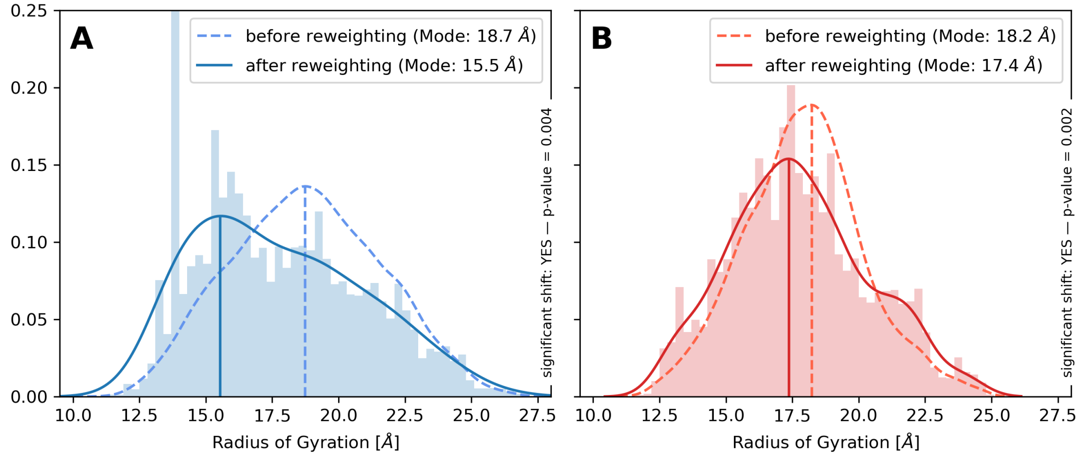

Figure S6: The kernel density estimation of the radius of gyration is shown for both the non-P (blue) and 4P peptide (red). Each plot shows the ensemble distribution of the radius of gyration before the reweighting (dashed blue line) and after the reweighting (solid line). Reweighting was based on the ^3J-values and the chemical shifts identified in Stöckelmaier et al. (PCCP 2024, 26, 23856). The reweighting strength was set to $\theta = 0.1$. The bars in the background represent the histogram of the reweighted ensemble. Repetition of figure 5 of the main manuscript for ease of comparison.

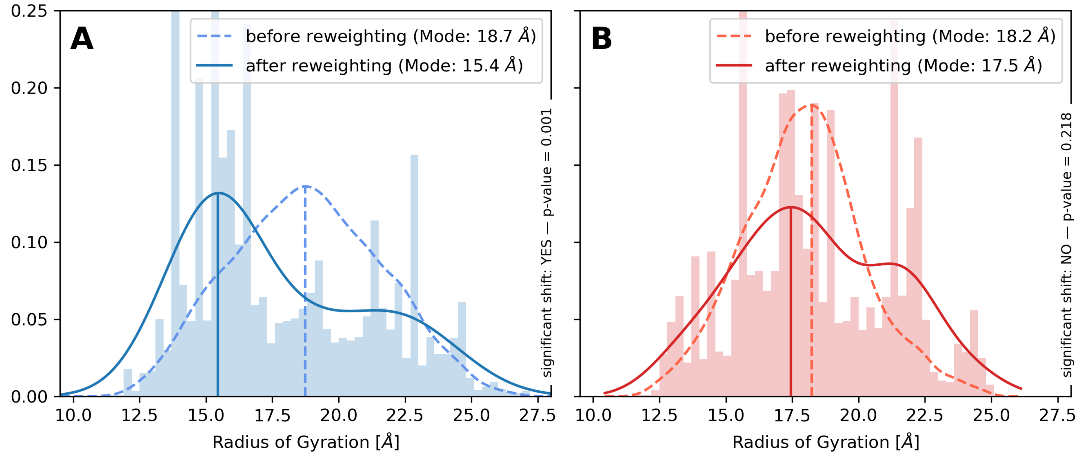

Figure S7: The kernel density estimation of the radius of gyration is shown for both the non-P (blue) and 4P peptide (red). Each plot shows the ensemble distribution of the radius of gyration before the reweighting (dashed blue line) and after the reweighting (solid line). Reweighting was based on the ^3J-values and the chemical shifts identified in Stöckelmaier et al. (PCCP 2024, 26, 23856). The reweighting strength was set to $\theta = 0.02$. The bars in the background represent the histogram of the reweighted ensemble.

4 DSSP

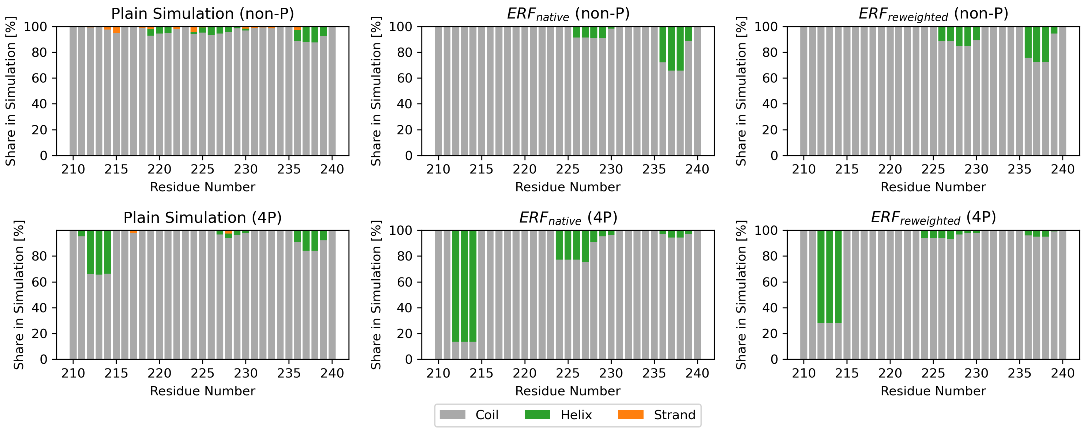

Figure S8: The analysis of the secondary structure of the conformers shows that the polypeptide remains in a coil-like conformation most of the time. Grey represents a coil-like secondary structure while green would suggest a helix-like one. *Helix* represents the sum of α-helix, π-helix and 3_{10}-helix; *Strand* represents an extended strand participating in a beta ladder (parallel and antiparallel). Figures labeled with ERF_{native} and $ERF_{reweighted}$ refer to the sparse ERF ensemble before and after reweighting, respectively. Reweighting was based on the ^3J-values and C_α and C_β chemical shifts. The reweighting strength was set to $\theta = 0.1$.

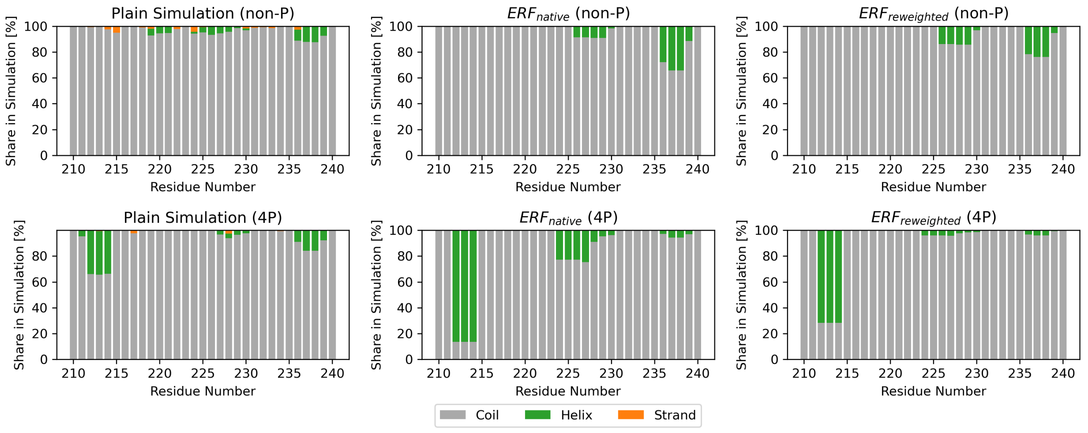

Figure S9: The analysis of the secondary structure of the conformers shows that the polypeptide remains in a coil-like conformation most of the time. Grey represents a coil-like secondary structure while green would suggest a helix-like one. *Helix* represents the sum of α-helix, π-helix and 3_{10}-helix; *Strand* represents an extended strand participating in a beta ladder (parallel and antiparallel). Figures labeled with ERF_{native} and $ERF_{reweighted}$ refer to the sparse ERF ensemble before and after reweighting, respectively. Reweighting was based on the ^3J-values and the chemical shifts identified in Stöckelmaier et al. (PCCP 2024, 26, 23856). The reweighting strength was set to $\theta = 0.1$. Repetition of figure 7 of the main manuscript, for ease of comparison.

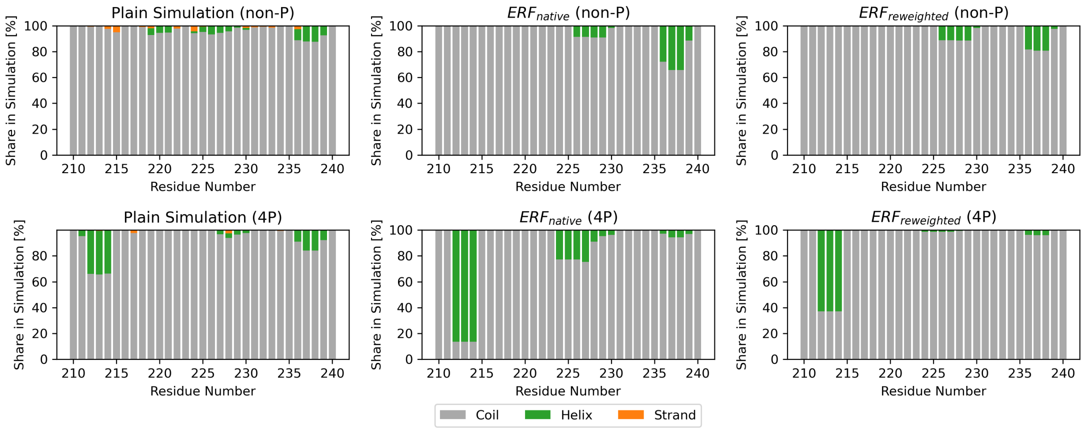

Figure S10: The analysis of the secondary structure of the conformers shows that the polypeptide remains in a coil-like conformation most of the time. Grey represents a coil-like secondary structure while green would suggest a helix-like one. *Helix* represents the sum of α-helix, π-helix and 3_{10}-helix; *Strand* represents an extended strand participating in a beta ladder (parallel and antiparallel). Figures labeled with ERF_{native} and $ERF_{reweighted}$ refer to the sparse ERF ensemble before and after reweighting, respectively. Reweighting was based on the ^{3}J-values and the chemical shifts identified in Stöckelmaier et al. (PCCP 2024, 26, 23856). The reweighting strength was set to $\theta = 0.02$.

5 The Kish Effective Sample Size

The Kish effective sample size has been calculated for all six ensemble reweightings and is summarized in Table 1.

Table 1: The Kish effective sample estimates the number of conformation, which significantly contribute to the ensemble properties after reweighting.

Ensemble	Reweighting Observables	θ	Conformations in Ensemble	Effective Sample Size (after reweighting)
non-P	3J_stoeckelmaier2024	0.02	24469	64
4P	3J_stoeckelmaier2024	0.02	12109	78
non-P	3J_stoeckelmaier2024	0.1	24469	158
4P	3J_stoeckelmaier2024	0.1	12109	985
non-P	3J_Ca_Cb	0.1	24469	1678
4P	3J_Ca_Cb	0.1	12109	2195

6 Convergence

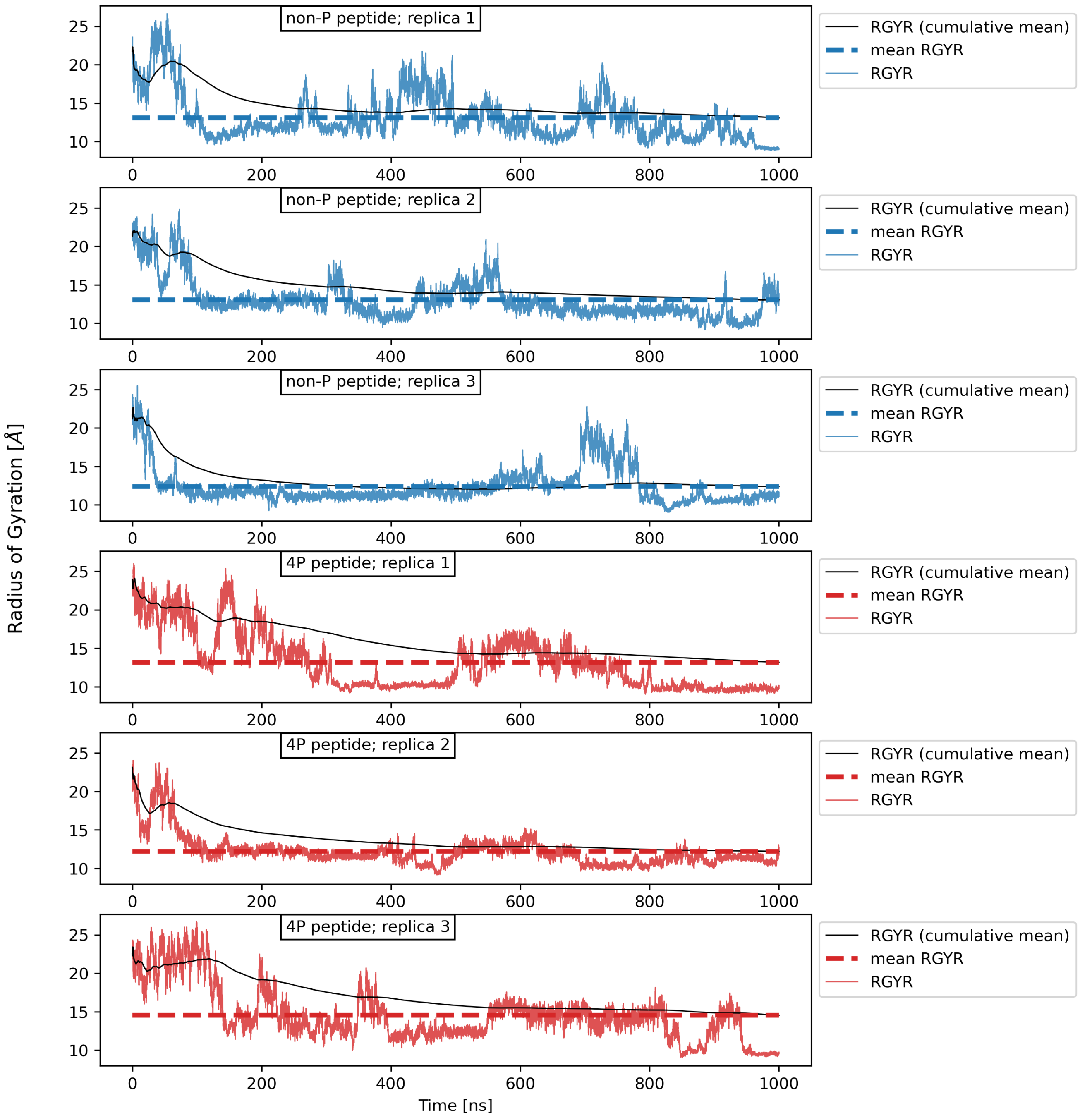

Figure S11: The solid black line represents the cumulative average of the radius of gyration (RGYR) from $time = 0$ to $time = t$ at each timestep t. At the end of the simulation the cumulative average is equal to the mean radius of gyration of the full simulation. A simulation might be interpreted as converged if the cumulative average of RGYR aligns with the value of the mean RGYR before the end of the simulation is reached. According to this metric, we consider replica 3 of the non-P simulation and replica 2 of the 4P simulation as converged; the other four replica are close to be converged.

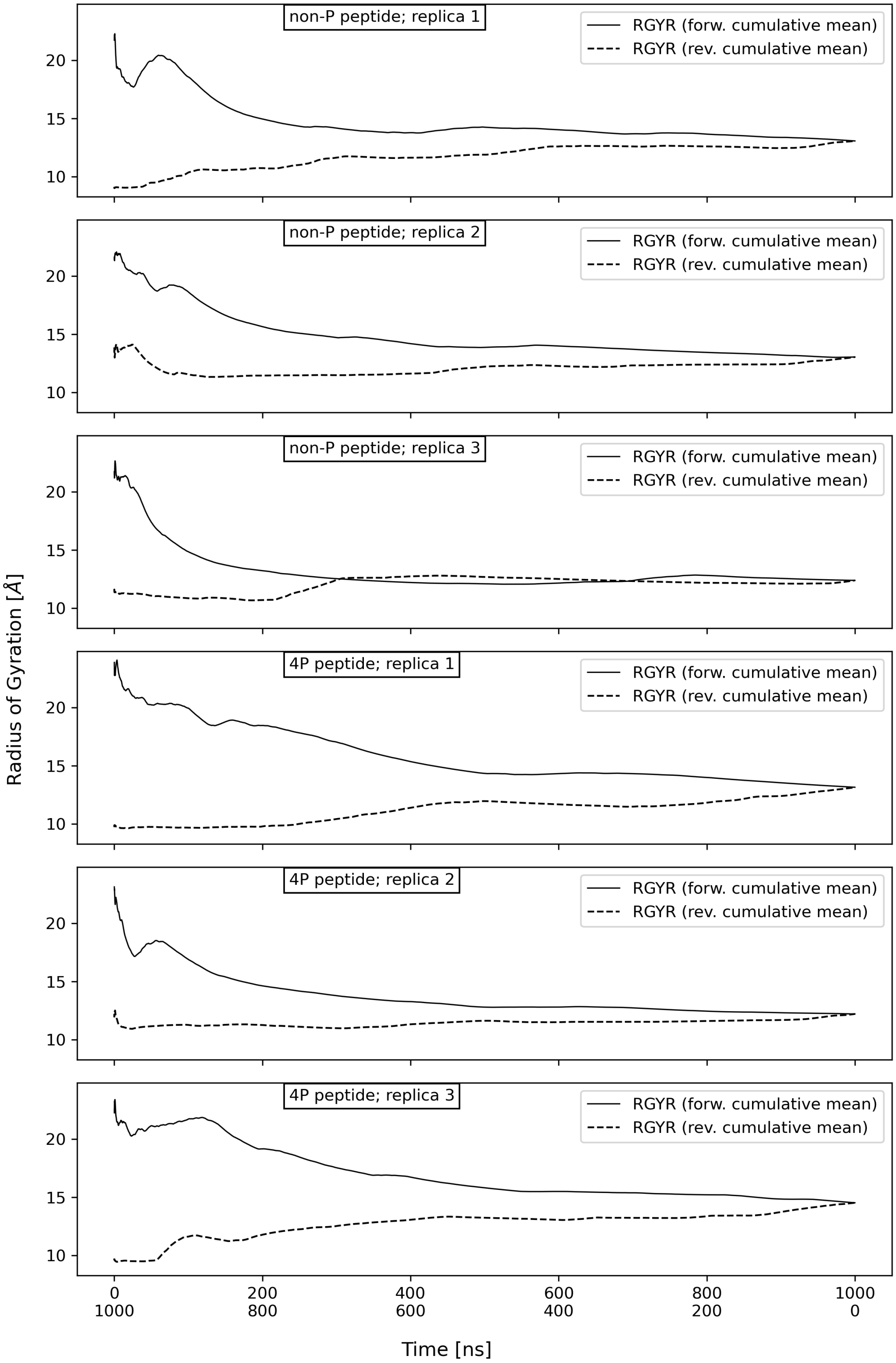

Figure S12: Similar to figure S11 the forward cumulative average of the radius of gyration (RGYR) is calculated (solid line). In addition, the reverse cumulative average RGYR is calculated, using increasing amounts of simulation data from the end of the simulation towards the beginning (inverted time, dashed line). A simulation might be considered as converged if the values of the forward and reverse direction align before the end of the simulation is reached. This is the case for replica 3 of the non-P peptide and very close for replica 2 of the 4P simulation, confirming the results of figure S11.

7 Individual per-Observable Error

In addition to Figure 2, which shows the root-mean-square deviation (RMSD) between the experimental data and simulated ensemble-averaged observables, the individual error between simulated and experimentally measured observables are presented here. For each of the six reweightings there is one table showing the error before and after the reweighting for both chemical shifts and ^3J-couplings.

7.1 Non-P Ensemble (Reweighting with C_α and C_β Chemical Shifts)

	Error [Hz] (native)	Error [Hz] (reweighted)
212_X_H_HA	2.12	0.75
214_X_H_HA	0.53	0.51
215_X_H_HA	0.45	0.09
217_X_H_HA	1.49	0.29
220_X_H_HA	1.04	0.12
222_X_H_HA	1.03	0.83
224_X_H_HA	0.17	0.17

	Error [Hz] (native)	Error [Hz] (reweighted)
226_X_H_HA	0.28	0.05
227_X_H_HA	0.13	0.14
228_X_H_HA	0.04	0.03
229_X_H_HA	1.58	0.63
230_X_H_HA	1.1	0.34
231_X_H_HA	1.6	0.64
234_X_H_HA	1.37	0.45

	Error [Hz] (native)	Error [Hz] (reweighted)
235_X_H_HA	0.96	0.19
237_X_H_HA	0.08	0.35
238_X_H_HA	0.67	0.49
239_X_H_HA	0.4	0.27
240_X_H_HA	0.51	0.21

Figure S13: The error between simulated and experimentally measured ^3J-coupling observables before and after reweighting, using ^3J-couplings and C_α and C_β chemical shifts. The reweighting strength was set to θ = 0.1.

	Error [ppm] (native)	Error [ppm] (reweighted)
210-SER-CA	0.73	0.75
210-SER-CB	0.37	0.34
210-SER-HA	0.25	0.26
210-SER-N	15.7	15.65
211-ARG-C	0.37	0.44
211-ARG-CA	0.14	0.26
211-ARG-CB	0.12	0.25
211-ARG-HA	0.05	0.0
211-ARG-N	0.45	0.44
212-THR-CA	0.11	0.07
212-THR-CB	0.23	0.28
212-THR-HN	0.4	0.4
212-THR-HA	0.12	0.09
212-THR-N	1.66	1.38
213-PRO-C	0.06	0.01
213-PRO-CA	0.01	0.09
213-PRO-CB	0.11	0.19
213-PRO-HA	0.06	0.06
214-SER-C	0.31	0.32
214-SER-CA	0.17	0.39
214-SER-CB	0.23	0.2
214-SER-HN	0.24	0.22
214-SER-HA	0.02	0.0
214-SER-N	1.1	1.02
215-LEU-CA	0.02	0.05
215-LEU-CB	0.03	0.05
215-LEU-HN	0.32	0.39
215-LEU-HA	0.05	0.04
215-LEU-N	1.3	1.91
216-PRO-C	0.16	0.1
216-PRO-CA	0.22	0.32
216-PRO-CB	0.21	0.16
216-PRO-HA	0.02	0.01
217-THR-CA	0.15	0.07
217-THR-CB	0.02	0.08
217-THR-HN	0.29	0.3
217-THR-HA	0.09	0.06
217-THR-N	0.7	0.63
218-PRO-CA	0.2	0.2
218-PRO-CB	0.3	0.32
218-PRO-HA	0.07	0.08
219-PRO-C	0.15	0.17
219-PRO-CA	0.12	0.15
219-PRO-CB	0.04	0.02
219-PRO-HA	0.03	0.03
220-THR-C	2.12	2.11
220-THR-CA	0.23	0.07
220-THR-CB	0.15	0.09
220-THR-HN	0.17	0.18
220-THR-HA	0.1	0.07
220-THR-N	0.09	0.05
221-ARG-C	0.04	0.03
221-ARG-CA	0.31	0.2

	Error [ppm] (native)	Error [ppm] (reweighted)
221-ARG-CB	0.16	0.22
221-ARG-HN	0.12	0.13
221-ARG-HA	0.0	0.03
221-ARG-N	0.16	0.06
222-GLU-CA	0.02	0.04
222-GLU-CB	0.14	0.15
222-GLU-HN	0.24	0.19
222-GLU-HA	0.19	0.19
222-GLU-N	1.88	1.63
223-PRO-C	0.17	0.24
223-PRO-CA	0.0	0.01
223-PRO-CB	0.16	0.14
223-PRO-HA	0.07	0.06
224-LYS-C	0.04	0.03
224-LYS-CA	0.24	0.29
224-LYS-CB	0.14	0.02
224-LYS-HN	0.16	0.16
224-LYS-HA	0.03	0.0
224-LYS-N	0.91	0.74
225-LYS-C	0.23	0.24
225-LYS-CA	0.41	0.46
225-LYS-CB	0.58	0.58
225-LYS-HN	0.11	0.09
225-LYS-HA	0.1	0.11
225-LYS-N	3.18	3.14
226-VAL-C	0.25	0.22
226-VAL-CA	0.47	0.5
226-VAL-CB	0.22	0.18
226-VAL-HN	0.28	0.27
226-VAL-HA	0.03	0.02
226-VAL-N	2.76	2.83
227-ALA-C	0.01	0.13
227-ALA-CA	0.44	0.47
227-ALA-CB	0.07	0.09
227-ALA-HN	0.21	0.2
227-ALA-HA	0.05	0.06
227-ALA-N	2.0	1.7
228-VAL-C	0.08	0.03
228-VAL-CA	0.09	0.24
228-VAL-CB	0.08	0.12
228-VAL-HN	0.17	0.18
228-VAL-HA	0.06	0.04
228-VAL-N	1.95	2.01
229-VAL-C	0.14	0.05
229-VAL-CA	0.13	0.22
229-VAL-CB	0.02	0.07
229-VAL-HN	0.32	0.3
229-VAL-HA	0.03	0.03
229-VAL-N	2.94	3.04
230-ARG-C	0.31	0.34
230-ARG-CA	0.14	0.28
230-ARG-CB	0.35	0.18
230-ARG-HN	0.44	0.48

	Error [ppm] (native)	Error [ppm] (reweighted)
230-ARG-HA	0.01	0.04
230-ARG-N	2.26	2.96
231-THR-CA	0.1	0.12
231-THR-CB	0.25	0.26
231-THR-HN	0.2	0.25
231-THR-HA	0.08	0.07
231-THR-N	1.44	1.62
232-PRO-CA	0.2	0.2
232-PRO-CB	0.21	0.21
232-PRO-HA	0.06	0.06
233-PRO-C	0.25	0.28
233-PRO-CA	0.13	0.09
233-PRO-CB	0.09	0.05
233-PRO-HA	0.03	0.04
234-LYS-C	0.22	0.24
234-LYS-CA	0.07	0.04
234-LYS-CB	0.07	0.11
234-LYS-HN	0.17	0.17
234-LYS-HA	0.04	0.02
234-LYS-N	0.72	0.59
235-SER-CA	0.14	0.1
235-SER-CB	0.36	0.37
235-SER-HN	0.32	0.29
235-SER-HA	0.1	0.08
235-SER-N	1.57	1.36
236-PRO-C	0.31	0.18
236-PRO-CA	0.43	0.43
236-PRO-CB	0.06	0.09
236-PRO-HA	0.05	0.05
237-SER-C	0.33	0.25
237-SER-CA	0.85	0.69
237-SER-CB	0.46	0.36
237-SER-HN	0.08	0.11
237-SER-HA	0.05	0.04
237-SER-N	0.52	0.46
238-SER-C	0.54	0.52
238-SER-CA	0.46	0.33
238-SER-CB	0.37	0.28
238-SER-HN	0.27	0.3
238-SER-HA	0.05	0.03
238-SER-N	1.51	1.5
239-ALA-C	0.66	0.72
239-ALA-CA	0.2	0.23
239-ALA-CB	0.02	0.04
239-ALA-HN	0.35	0.31
239-ALA-HA	0.06	0.06
239-ALA-N	1.34	1.18
240-LYS-CA	0.35	0.36
240-LYS-CB	0.06	0.13
240-LYS-HN	0.04	0.03
240-LYS-HA	0.04	0.03
240-LYS-N	3.92	3.94

Figure S14: The error between simulated and experimentally measured chemical shift observables before and after reweighting, using ^3J-couplings and C_α and C_β chemical shifts. The reweighting strength was set to θ = 0.1.

7.2 4P Ensemble (Reweighting with C_α and C_β Chemical Shifts)

	Error [Hz] (native)	Error [Hz] (reweighted)
212_X_H_HA	0.9	0.48
214_X_H_HA	0.44	0.24
215_X_H_HA	0.78	0.23
217_X_H_HA	0.09	0.02
220_X_H_HA	0.22	0.13
222_X_H_HA	1.61	0.82
224_X_H_HA	1.03	0.36

	Error [Hz] (native)	Error [Hz] (reweighted)
226_X_H_HA	0.63	0.24
227_X_H_HA	0.35	0.06
228_X_H_HA	0.95	0.2
229_X_H_HA	1.42	0.45
230_X_H_HA	0.79	0.16
231_X_H_HA	0.96	0.36
234_X_H_HA	0.11	0.1

	Error [Hz] (native)	Error [Hz] (reweighted)
235_X_H_HA	1.24	0.65
237_X_H_HA	0.23	0.08
238_X_H_HA	0.18	0.12
239_X_H_HA	0.42	0.18
240_X_H_HA	0.34	0.04

Figure S15: The error between simulated and experimentally measured ^3J-coupling observables before and after reweighting, using ^3J-couplings and C_α and C_β chemical shifts. The reweighting strength was set to $\theta = 0.1$.

	Error [ppm] (native)	Error [ppm] (reweighted)
210-SER-CA	0.63	0.68
210-SER-CB	0.71	0.7
210-SER-HA	0.23	0.23
211-ARG-CA	0.07	0.15
211-ARG-CB	0.81	0.65
211-ARG-HA	0.11	0.09
211-ARG-N	3.07	2.95
213-PRO-C	0.85	0.65
213-PRO-CA	1.52	1.2
213-PRO-CB	0.67	0.45
213-PRO-HA	0.0	0.01
214-SER-C	0.73	0.69
214-SER-CA	0.6	0.78
214-SER-CB	0.07	0.14
214-SER-HN	0.43	0.38
214-SER-HA	0.0	0.01
214-SER-N	3.73	3.5
215-LEU-HN	0.88	0.87
215-LEU-HA	0.08	0.05
215-LEU-N	1.62	2.07
216-PRO-C	0.95	0.97
216-PRO-CA	0.02	0.05
216-PRO-CB	0.25	0.24
216-PRO-HA	0.02	0.01
218-PRO-CA	0.3	0.31
218-PRO-CB	0.18	0.2
218-PRO-HA	0.09	0.1
219-PRO-C	0.05	0.07
219-PRO-CA	0.21	0.2
219-PRO-CB	0.01	0.04
219-PRO-HA	0.04	0.03
220-THR-C	2.15	2.15
220-THR-CA	0.14	0.2
220-THR-CB	0.05	0.03
220-THR-HN	0.1	0.11
220-THR-HA	0.1	0.1
220-THR-N	0.08	0.08
221-ARG-C	0.1	0.03
221-ARG-CA	0.25	0.51
221-ARG-CB	0.14	0.02
221-ARG-HN	0.11	0.06
221-ARG-HA	0.03	0.02
221-ARG-N	0.63	0.68
222-GLU-CA	0.11	0.09
222-GLU-CB	0.23	0.22

	Error [ppm] (native)	Error [ppm] (reweighted)
222-GLU-HN	0.19	0.25
222-GLU-HA	0.18	0.17
222-GLU-N	1.7	2.03
223-PRO-C	0.38	0.25
223-PRO-CA	0.0	0.09
223-PRO-CB	0.15	0.17
223-PRO-HA	0.07	0.07
224-LYS-C	0.4	0.07
224-LYS-CA	1.06	0.39
224-LYS-CB	0.06	0.03
224-LYS-HN	0.04	0.11
224-LYS-HA	0.03	0.04
224-LYS-N	0.02	0.21
225-LYS-C	0.16	0.13
225-LYS-CA	0.76	0.45
225-LYS-CB	0.11	0.08
225-LYS-HN	0.07	0.14
225-LYS-HA	0.08	0.08
225-LYS-N	3.1	2.49
226-VAL-C	0.6	0.32
226-VAL-CA	0.54	0.07
226-VAL-CB	0.12	0.0
226-VAL-HN	0.25	0.19
226-VAL-HA	0.03	0.09
226-VAL-N	2.5	2.0
227-ALA-C	0.32	0.04
227-ALA-CA	0.72	0.29
227-ALA-CB	0.01	0.18
227-ALA-HN	0.33	0.29
227-ALA-HA	0.05	0.01
227-ALA-N	2.51	1.55
228-VAL-C	0.02	0.1
228-VAL-CA	0.28	0.0
228-VAL-CB	0.19	0.06
228-VAL-HN	0.27	0.13
228-VAL-HA	0.07	0.11
228-VAL-N	3.08	2.48
229-VAL-C	0.09	0.0
229-VAL-CA	0.19	0.18
229-VAL-CB	0.27	0.02
229-VAL-HN	0.43	0.36
229-VAL-HA	0.05	0.11
229-VAL-N	3.91	3.89
230-ARG-C	0.28	0.31
230-ARG-CA	0.6	0.69

	Error [ppm] (native)	Error [ppm] (reweighted)
230-ARG-CB	0.27	0.14
230-ARG-HN	0.25	0.19
230-ARG-HA	0.03	0.01
230-ARG-N	2.11	2.23
232-PRO-CA	0.31	0.31
232-PRO-CB	0.24	0.24
232-PRO-HA	0.07	0.07
233-PRO-C	0.26	0.26
233-PRO-CA	0.25	0.23
233-PRO-CB	0.04	0.02
233-PRO-HA	0.03	0.03
234-LYS-C	0.12	0.07
234-LYS-CA	0.34	0.39
234-LYS-CB	0.13	0.16
234-LYS-HN	0.19	0.18
234-LYS-HA	0.01	0.0
234-LYS-N	0.69	0.57
236-PRO-C	0.35	0.41
236-PRO-CA	0.5	0.39
236-PRO-CB	0.14	0.11
236-PRO-HA	0.01	0.0
237-SER-C	0.14	0.2
237-SER-CA	0.14	0.24
237-SER-CB	0.33	0.34
237-SER-HN	0.04	0.07
237-SER-HA	0.01	0.02
237-SER-N	0.58	0.39
238-SER-C	0.5	0.43
238-SER-CA	0.07	0.09
238-SER-CB	0.02	0.09
238-SER-HN	0.08	0.08
238-SER-HA	0.01	0.02
238-SER-N	0.4	0.25
239-ALA-C	0.69	0.68
239-ALA-CA	0.1	0.1
239-ALA-CB	0.03	0.04
239-ALA-HN	0.07	0.07
239-ALA-HA	0.06	0.06
239-ALA-N	0.43	0.23
240-LYS-CA	0.37	0.37
240-LYS-CB	0.17	0.22
240-LYS-HN	0.02	0.03
240-LYS-HA	0.09	0.1
240-LYS-N	3.41	3.28

Figure S16: The error between simulated and experimentally measured chemical shift observables before and after reweighting, using ^3J-couplings and C_α and C_β chemical shifts. The reweighting strength was set to θ = 0.1.

7.3 Non-P Ensemble (Reweighting with Stöckelmaier et al. identified Chemical Shifts and $\theta = 0.1$)

	Error [Hz] (native)	Error [Hz] (reweighted)
212_X_H_HA	2.12	0.78
214_X_H_HA	0.53	0.51
215_X_H_HA	0.45	0.17
217_X_H_HA	1.49	0.24
220_X_H_HA	1.04	0.01
222_X_H_HA	1.03	0.75
224_X_H_HA	0.17	0.1

	Error [Hz] (native)	Error [Hz] (reweighted)
226_X_H_HA	0.28	0.31
227_X_H_HA	0.13	0.01
228_X_H_HA	0.04	0.1
229_X_H_HA	1.58	0.48
230_X_H_HA	1.1	0.18
231_X_H_HA	1.6	0.54
234_X_H_HA	1.37	0.45

	Error [Hz] (native)	Error [Hz] (reweighted)
235_X_H_HA	0.96	0.26
237_X_H_HA	0.08	0.17
238_X_H_HA	0.67	0.35
239_X_H_HA	0.4	0.28
240_X_H_HA	0.51	0.22

Figure S17: The error between simulated and experimentally measured ^3J-coupling observables before and after reweighting, using ^3J-couplings and the chemical shifts identified in Stöckelmaier et al. (PCCP 2024, 26, 23856). The reweighting strength was set to $\theta = 0.1$.

	Error [ppm] (native)	Error [ppm] (reweighted)
210-SER-CA	0.73	0.79
210-SER-CB	0.37	0.37
210-SER-HA	0.25	0.25
210-SER-N	15.7	15.66
211-ARG-C	0.37	0.42
211-ARG-CA	0.14	0.32
211-ARG-CB	0.12	0.33
211-ARG-HA	0.05	0.01
211-ARG-N	0.45	0.36
212-THR-CA	0.11	0.11
212-THR-CB	0.23	0.27
212-THR-HN	0.4	0.42
212-THR-HA	0.12	0.1
212-THR-N	1.66	1.22
213-PRO-C	0.06	0.01
213-PRO-CA	0.01	0.05
213-PRO-CB	0.11	0.17
213-PRO-HA	0.06	0.06
214-SER-C	0.31	0.36
214-SER-CA	0.17	0.37
214-SER-CB	0.23	0.23
214-SER-HN	0.24	0.24
214-SER-HA	0.02	0.0
214-SER-N	1.1	1.03
215-LEU-CA	0.02	0.06
215-LEU-CB	0.03	0.0
215-LEU-HN	0.32	0.37
215-LEU-HA	0.05	0.05
215-LEU-N	1.3	1.8
216-PRO-C	0.16	0.07
216-PRO-CA	0.22	0.3
216-PRO-CB	0.21	0.17
216-PRO-HA	0.02	0.0
217-THR-CA	0.15	0.07
217-THR-CB	0.02	0.07
217-THR-HN	0.29	0.25
217-THR-HA	0.09	0.05
217-THR-N	0.7	0.47
218-PRO-CA	0.2	0.19
218-PRO-CB	0.3	0.31
218-PRO-HA	0.07	0.07
219-PRO-C	0.15	0.11
219-PRO-CA	0.12	0.13
219-PRO-CB	0.04	0.02
219-PRO-HA	0.03	0.03
220-THR-C	2.12	2.07
220-THR-CA	0.23	0.01
220-THR-CB	0.15	0.09
220-THR-HN	0.17	0.18
220-THR-HA	0.1	0.05
220-THR-N	0.09	0.05
221-ARG-C	0.04	0.02
221-ARG-CA	0.31	0.18
221-ARG-CB	0.16	0.23
221-ARG-HN	0.12	0.12
221-ARG-HA	0.0	0.03
221-ARG-N	0.16	0.15
222-GLU-CA	0.02	0.05
222-GLU-CB	0.14	0.19
222-GLU-HN	0.24	0.18
222-GLU-HA	0.19	0.19
222-GLU-N	1.88	1.28
223-PRO-C	0.17	0.28
223-PRO-CA	0.0	0.01
223-PRO-CB	0.16	0.11
223-PRO-HA	0.07	0.06
224-LYS-C	0.04	0.05
224-LYS-CA	0.24	0.31
224-LYS-CB	0.14	0.01
224-LYS-HN	0.16	0.15
224-LYS-HA	0.03	0.0
224-LYS-N	0.91	0.62
225-LYS-C	0.23	0.24
225-LYS-CA	0.41	0.49
225-LYS-CB	0.58	0.63
225-LYS-HN	0.11	0.07
225-LYS-HA	0.1	0.11
225-LYS-N	3.18	2.93
226-VAL-C	0.25	0.3
226-VAL-CA	0.47	0.56
226-VAL-CB	0.22	0.25
226-VAL-HN	0.28	0.29
226-VAL-HA	0.03	0.0
226-VAL-N	2.76	2.21
227-ALA-C	0.01	0.14
227-ALA-CA	0.44	0.53
227-ALA-CB	0.07	0.02
227-ALA-HN	0.21	0.18
227-ALA-HA	0.05	0.06
227-ALA-N	2.0	1.57
228-VAL-C	0.08	0.01
228-VAL-CA	0.09	0.16
228-VAL-CB	0.08	0.06
228-VAL-HN	0.17	0.21
228-VAL-HA	0.06	0.07
228-VAL-N	1.95	2.17
229-VAL-C	0.14	0.09
229-VAL-CA	0.13	0.24
229-VAL-CB	0.02	0.02
229-VAL-HN	0.32	0.24
229-VAL-HA	0.03	0.03
229-VAL-N	2.94	2.61
230-ARG-C	0.31	0.35
230-ARG-CA	0.14	0.28
230-ARG-CB	0.35	0.24
230-ARG-HN	0.44	0.45
230-ARG-HA	0.01	0.04
230-ARG-N	2.26	2.76
231-THR-CA	0.1	0.25
231-THR-CB	0.25	0.25
231-THR-HN	0.2	0.22
231-THR-HA	0.08	0.08
231-THR-N	1.44	1.25
232-PRO-CA	0.2	0.19
232-PRO-CB	0.21	0.22
232-PRO-HA	0.06	0.06
233-PRO-C	0.25	0.34
233-PRO-CA	0.13	0.09
233-PRO-CB	0.09	0.05
233-PRO-HA	0.03	0.04
234-LYS-C	0.22	0.24
234-LYS-CA	0.07	0.1
234-LYS-CB	0.07	0.03
234-LYS-HN	0.17	0.18
234-LYS-HA	0.04	0.04
234-LYS-N	0.72	0.64
235-SER-CA	0.14	0.04
235-SER-CB	0.36	0.24
235-SER-HN	0.32	0.24
235-SER-HA	0.1	0.09
235-SER-N	1.57	1.27
236-PRO-C	0.31	0.15
236-PRO-CA	0.43	0.5
236-PRO-CB	0.06	0.09
236-PRO-HA	0.05	0.07
237-SER-C	0.33	0.17
237-SER-CA	0.85	0.45
237-SER-CB	0.46	0.29
237-SER-HN	0.08	0.1
237-SER-HA	0.05	0.02
237-SER-N	0.52	0.63
238-SER-C	0.54	0.64
238-SER-CA	0.46	0.2
238-SER-CB	0.37	0.19
238-SER-HN	0.27	0.28
238-SER-HA	0.05	0.01
238-SER-N	1.51	1.05
239-ALA-C	0.66	0.73
239-ALA-CA	0.2	0.22
239-ALA-CB	0.02	0.05
239-ALA-HN	0.35	0.24
239-ALA-HA	0.06	0.07
239-ALA-N	1.34	0.76
240-LYS-CA	0.35	0.39
240-LYS-CB	0.06	0.21
240-LYS-HN	0.04	0.0
240-LYS-HA	0.04	0.04
240-LYS-N	3.92	3.79

Figure S18: The error between simulated and experimentally measured chemical shift observables before and after reweighting, using ^3J-couplings and the chemical shifts identified in Stöckelmaier et al. (PCCP 2024, 26, 23856). The reweighting strength was set to $\theta = 0.1$.

7.4 4P Ensemble (Reweighting with Stöckelmaier et al. identified Chemical Shifts and $\theta = 0.1$)

	Error [Hz] (native)	Error [Hz] (reweighted)
212_X_H_HA	0.9	0.59
214_X_H_HA	0.44	0.25
215_X_H_HA	0.78	0.25
217_X_H_HA	0.09	0.04
220_X_H_HA	0.22	0.15
222_X_H_HA	1.61	0.91
224_X_H_HA	1.03	0.15

	Error [Hz] (native)	Error [Hz] (reweighted)
226_X_H_HA	0.63	0.09
227_X_H_HA	0.35	0.06
228_X_H_HA	0.95	0.28
229_X_H_HA	1.42	0.29
230_X_H_HA	0.79	0.14
231_X_H_HA	0.96	0.31
234_X_H_HA	0.11	0.16

	Error [Hz] (native)	Error [Hz] (reweighted)
235_X_H_HA	1.24	0.56
237_X_H_HA	0.23	0.03
238_X_H_HA	0.18	0.06
239_X_H_HA	0.42	0.08
240_X_H_HA	0.34	0.05

Figure S19: The error between simulated and experimentally measured ^3J-coupling observables before and after reweighting, using ^3J-couplings and the chemical shifts identified in Stöckelmaier et al. (PCCP 2024, 26, 23856). The reweighting strength was set to $\theta = 0.1$.

	Error [ppm] (native)	Error [ppm] (reweighted)
210-SER-CA	0.63	0.69
210-SER-CB	0.71	0.7
210-SER-HA	0.23	0.23
211-ARG-CA	0.07	0.12
211-ARG-CB	0.81	0.74
211-ARG-HA	0.11	0.1
211-ARG-N	3.07	2.97
213-PRO-C	0.85	0.69
213-PRO-CA	1.52	1.26
213-PRO-CB	0.67	0.48
213-PRO-HA	0.0	0.01
214-SER-C	0.73	0.69
214-SER-CA	0.6	0.76
214-SER-CB	0.07	0.13
214-SER-HN	0.43	0.37
214-SER-HA	0.0	0.01
214-SER-N	3.73	3.55
215-LEU-HN	0.88	0.87
215-LEU-HA	0.08	0.05
215-LEU-N	1.62	2.06
216-PRO-C	0.95	0.97
216-PRO-CA	0.02	0.07
216-PRO-CB	0.25	0.23
216-PRO-HA	0.02	0.01
218-PRO-CA	0.3	0.31
218-PRO-CB	0.18	0.19
218-PRO-HA	0.09	0.1
219-PRO-C	0.05	0.12
219-PRO-CA	0.21	0.27
219-PRO-CB	0.01	0.03
219-PRO-HA	0.04	0.04
220-THR-C	2.15	2.28
220-THR-CA	0.14	0.22
220-THR-CB	0.05	0.06
220-THR-HN	0.1	0.11
220-THR-HA	0.1	0.11
220-THR-N	0.08	0.21
221-ARG-C	0.1	0.03
221-ARG-CA	0.25	0.35
221-ARG-CB	0.14	0.1
221-ARG-HN	0.11	0.09
221-ARG-HA	0.03	0.0
221-ARG-N	0.63	0.69
222-GLU-CA	0.11	0.09
222-GLU-CB	0.23	0.21
222-GLU-HN	0.19	0.18
222-GLU-HA	0.18	0.16
222-GLU-N	1.7	1.79
223-PRO-C	0.38	0.31
223-PRO-CA	0.0	0.15
223-PRO-CB	0.15	0.14
223-PRO-HA	0.07	0.07
224-LYS-C	0.4	0.04
224-LYS-CA	1.06	0.22
224-LYS-CB	0.06	0.12
224-LYS-HN	0.04	0.14
224-LYS-HA	0.03	0.05
224-LYS-N	0.02	0.22
225-LYS-C	0.16	0.08
225-LYS-CA	0.76	0.36
225-LYS-CB	0.11	0.01
225-LYS-HN	0.07	0.11
225-LYS-HA	0.08	0.08
225-LYS-N	3.1	1.98
226-VAL-C	0.6	0.3
226-VAL-CA	0.54	0.12
226-VAL-CB	0.12	0.07
226-VAL-HN	0.25	0.15
226-VAL-HA	0.03	0.09
226-VAL-N	2.5	1.69
227-ALA-C	0.32	0.15
227-ALA-CA	0.72	0.18
227-ALA-CB	0.01	0.21
227-ALA-HN	0.33	0.31
227-ALA-HA	0.05	0.01
227-ALA-N	2.51	1.69
228-VAL-C	0.02	0.16
228-VAL-CA	0.28	0.06
228-VAL-CB	0.19	0.04
228-VAL-HN	0.27	0.13
228-VAL-HA	0.07	0.1
228-VAL-N	3.08	2.23
229-VAL-C	0.09	0.02
229-VAL-CA	0.19	0.09
229-VAL-CB	0.27	0.09
229-VAL-HN	0.43	0.26
229-VAL-HA	0.05	0.1
229-VAL-N	3.91	3.04
230-ARG-C	0.28	0.3
230-ARG-CA	0.6	0.69
230-ARG-CB	0.27	0.17
230-ARG-HN	0.25	0.19
230-ARG-HA	0.03	0.01
230-ARG-N	2.11	2.06
232-PRO-CA	0.31	0.3
232-PRO-CB	0.24	0.24
232-PRO-HA	0.07	0.07
233-PRO-C	0.26	0.24
233-PRO-CA	0.25	0.23
233-PRO-CB	0.04	0.02
233-PRO-HA	0.03	0.03
234-LYS-C	0.12	0.05
234-LYS-CA	0.34	0.44
234-LYS-CB	0.13	0.17
234-LYS-HN	0.19	0.17
234-LYS-HA	0.01	0.01
234-LYS-N	0.69	0.49
236-PRO-C	0.35	0.4
236-PRO-CA	0.5	0.39
236-PRO-CB	0.14	0.1
236-PRO-HA	0.01	0.0
237-SER-C	0.14	0.24
237-SER-CA	0.14	0.26
237-SER-CB	0.33	0.33
237-SER-HN	0.04	0.07
237-SER-HA	0.01	0.02
237-SER-N	0.58	0.34
238-SER-C	0.5	0.4
238-SER-CA	0.07	0.09
238-SER-CB	0.02	0.11
238-SER-HN	0.08	0.09
238-SER-HA	0.01	0.03
238-SER-N	0.4	0.28
239-ALA-C	0.69	0.7
239-ALA-CA	0.1	0.09
239-ALA-CB	0.03	0.06
239-ALA-HN	0.07	0.06
239-ALA-HA	0.06	0.06
239-ALA-N	0.43	0.19
240-LYS-CA	0.37	0.37
240-LYS-CB	0.17	0.21
240-LYS-HN	0.02	0.03
240-LYS-HA	0.09	0.09
240-LYS-N	3.41	3.25

Figure S20: The error between simulated and experimentally measured chemical shift observables before and after reweighting, using ^3J-couplings and the chemical shifts identified in Stöckelmaier et al. (PCCP 2024, 26, 23856). The reweighting strength was set to $\theta = 0.1$.

7.5 Non-P Ensemble (Reweighting with Stöckelmaier et al. identified Chemical Shifts and $\theta = 0.02$)

	Error [Hz] (native)	Error [Hz] (reweighted)
212_X_H_HA	2.12	0.45
214_X_H_HA	0.53	0.31
215_X_H_HA	0.45	0.17
217_X_H_HA	1.49	0.05
220_X_H_HA	1.04	0.09
222_X_H_HA	1.03	0.44
224_X_H_HA	0.17	0.02

	Error [Hz] (native)	Error [Hz] (reweighted)
226_X_H_HA	0.28	0.06
227_X_H_HA	0.13	0.2
228_X_H_HA	0.04	0.01
229_X_H_HA	1.58	0.16
230_X_H_HA	1.1	0.05
231_X_H_HA	1.6	0.19
234_X_H_HA	1.37	0.21

	Error [Hz] (native)	Error [Hz] (reweighted)
235_X_H_HA	0.96	0.14
237_X_H_HA	0.08	0.05
238_X_H_HA	0.67	0.03
239_X_H_HA	0.4	0.06
240_X_H_HA	0.51	0.14

Figure S21: The error between simulated and experimentally measured ^3J-coupling observables before and after reweighting, using ^3J-couplings and the chemical shifts identified in Stöckelmaier et al. (PCCP 2024, 26, 23856). The reweighting strength was set to $\theta = 0.02$.

	Error [ppm] (native)	Error [ppm] (reweighted)
210-SER-CA	0.73	0.71
210-SER-CB	0.37	0.38
210-SER-HA	0.25	0.25
210-SER-N	15.7	15.65
211-ARG-C	0.37	0.41
211-ARG-CA	0.14	0.34
211-ARG-CB	0.12	0.33
211-ARG-HA	0.05	0.01
211-ARG-N	0.45	0.4
212-THR-CA	0.11	0.11
212-THR-CB	0.23	0.28
212-THR-HN	0.4	0.38
212-THR-HA	0.12	0.1
212-THR-N	1.66	1.12
213-PRO-C	0.06	0.0
213-PRO-CA	0.01	0.01
213-PRO-CB	0.11	0.16
213-PRO-HA	0.06	0.06
214-SER-C	0.31	0.36
214-SER-CA	0.17	0.4
214-SER-CB	0.23	0.3
214-SER-HN	0.24	0.24
214-SER-HA	0.02	0.01
214-SER-N	1.1	1.17
215-LEU-CA	0.02	0.01
215-LEU-CB	0.03	0.02
215-LEU-HN	0.32	0.41
215-LEU-HA	0.05	0.05
215-LEU-N	1.3	1.81
216-PRO-C	0.16	0.08
216-PRO-CA	0.22	0.37
216-PRO-CB	0.21	0.15
216-PRO-HA	0.02	0.0
217-THR-CA	0.15	0.05
217-THR-CB	0.02	0.05
217-THR-HN	0.29	0.32
217-THR-HA	0.09	0.05
217-THR-N	0.7	1.01
218-PRO-CA	0.2	0.17
218-PRO-CB	0.3	0.29
218-PRO-HA	0.07	0.08
219-PRO-C	0.15	0.12
219-PRO-CA	0.12	0.09
219-PRO-CB	0.04	0.09
219-PRO-HA	0.03	0.02
220-THR-C	2.12	2.13
220-THR-CA	0.23	0.0
220-THR-CB	0.15	0.18
220-THR-HN	0.17	0.2
220-THR-HA	0.1	0.06
220-THR-N	0.09	0.06
221-ARG-C	0.04	0.08
221-ARG-CA	0.31	0.17

	Error [ppm] (native)	Error [ppm] (reweighted)
221-ARG-CB	0.16	0.22
221-ARG-HN	0.12	0.12
221-ARG-HA	0.0	0.02
221-ARG-N	0.16	0.4
222-GLU-CA	0.02	0.11
222-GLU-CB	0.14	0.22
222-GLU-HN	0.24	0.13
222-GLU-HA	0.19	0.18
222-GLU-N	1.88	0.92
223-PRO-C	0.17	0.38
223-PRO-CA	0.0	0.04
223-PRO-CB	0.16	0.07
223-PRO-HA	0.07	0.05
224-LYS-C	0.04	0.07
224-LYS-CA	0.24	0.3
224-LYS-CB	0.14	0.04
224-LYS-HN	0.16	0.13
224-LYS-HA	0.03	0.01
224-LYS-N	0.91	0.32
225-LYS-C	0.23	0.26
225-LYS-CA	0.41	0.57
225-LYS-CB	0.58	0.61
225-LYS-HN	0.11	0.04
225-LYS-HA	0.1	0.12
225-LYS-N	3.18	2.43
226-VAL-C	0.25	0.24
226-VAL-CA	0.47	0.45
226-VAL-CB	0.22	0.25
226-VAL-HN	0.28	0.3
226-VAL-HA	0.03	0.01
226-VAL-N	2.76	2.09
227-ALA-C	0.01	0.05
227-ALA-CA	0.44	0.46
227-ALA-CB	0.07	0.07
227-ALA-HN	0.21	0.2
227-ALA-HA	0.05	0.05
227-ALA-N	2.0	1.17
228-VAL-C	0.08	0.19
228-VAL-CA	0.09	0.01
228-VAL-CB	0.08	0.08
228-VAL-HN	0.17	0.2
228-VAL-HA	0.06	0.08
228-VAL-N	1.95	2.07
229-VAL-C	0.14	0.14
229-VAL-CA	0.13	0.15
229-VAL-CB	0.02	0.11
229-VAL-HN	0.32	0.19
229-VAL-HA	0.03	0.05
229-VAL-N	2.94	2.22
230-ARG-C	0.31	0.33
230-ARG-CA	0.14	0.3
230-ARG-CB	0.35	0.25
230-ARG-HN	0.44	0.41

	Error [ppm] (native)	Error [ppm] (reweighted)
230-ARG-HA	0.01	0.04
230-ARG-N	2.26	2.38
231-THR-CA	0.1	0.3
231-THR-CB	0.25	0.24
231-THR-HN	0.2	0.22
231-THR-HA	0.08	0.09
231-THR-N	1.44	0.84
232-PRO-CA	0.2	0.18
232-PRO-CB	0.21	0.24
232-PRO-HA	0.06	0.06
233-PRO-C	0.25	0.39
233-PRO-CA	0.13	0.06
233-PRO-CB	0.09	0.06
233-PRO-HA	0.03	0.04
234-LYS-C	0.22	0.26
234-LYS-CA	0.07	0.17
234-LYS-CB	0.07	0.02
234-LYS-HN	0.17	0.18
234-LYS-HA	0.04	0.04
234-LYS-N	0.72	0.6
235-SER-CA	0.14	0.02
235-SER-CB	0.36	0.2
235-SER-HN	0.32	0.2
235-SER-HA	0.1	0.09
235-SER-N	1.57	1.11
236-PRO-C	0.31	0.09
236-PRO-CA	0.43	0.48
236-PRO-CB	0.06	0.09
236-PRO-HA	0.05	0.07
237-SER-C	0.33	0.08
237-SER-CA	0.85	0.23
237-SER-CB	0.46	0.19
237-SER-HN	0.08	0.12
237-SER-HA	0.05	0.0
237-SER-N	0.52	0.58
238-SER-C	0.54	0.71
238-SER-CA	0.46	0.08
238-SER-CB	0.37	0.08
238-SER-HN	0.27	0.27
238-SER-HA	0.05	0.01
238-SER-N	1.51	0.75
239-ALA-C	0.66	0.78
239-ALA-CA	0.2	0.26
239-ALA-CB	0.02	0.1
239-ALA-HN	0.35	0.17
239-ALA-HA	0.06	0.08
239-ALA-N	1.34	0.51
240-LYS-CA	0.35	0.41
240-LYS-CB	0.06	0.31
240-LYS-HN	0.04	0.0
240-LYS-HA	0.04	0.04
240-LYS-N	3.92	3.91

Figure S22: The error between simulated and experimentally measured chemical shift observables before and after reweighting, using ^3J-couplings and the chemical shifts identified in Stöckelmaier et al. (PCCP 2024, 26, 23856). The reweighting strength was set to $\theta = 0.02$.

7.6 4P Ensemble (Reweighting with Stöckelmaier et al. identified Chemical Shifts and $\theta = 0.02$)

	Error [Hz] (native)	Error [Hz] (reweighted)
212_X_H_HA	0.9	0.15
214_X_H_HA	0.44	0.08
215_X_H_HA	0.78	0.08
217_X_H_HA	0.09	0.08
220_X_H_HA	0.22	0.06
222_X_H_HA	1.61	0.47
224_X_H_HA	1.03	0.07

	Error [Hz] (native)	Error [Hz] (reweighted)
226_X_H_HA	0.63	0.06
227_X_H_HA	0.35	0.04
228_X_H_HA	0.95	0.19
229_X_H_HA	1.42	0.11
230_X_H_HA	0.79	0.09
231_X_H_HA	0.96	0.12
234_X_H_HA	0.11	0.13

	Error [Hz] (native)	Error [Hz] (reweighted)
235_X_H_HA	1.24	0.24
237_X_H_HA	0.23	0.06
238_X_H_HA	0.18	0.04
239_X_H_HA	0.42	0.03
240_X_H_HA	0.34	0.04

Figure S23: The error between simulated and experimentally measured ^3J-coupling observables before and after reweighting, using ^3J-couplings and the chemical shifts identified in Stöckelmaier et al. (PCCP 2024, 26, 23856). The reweighting strength was set to $\theta = 0.02$.

	Error [ppm] (native)	Error [ppm] (reweighted)
210-SER-CA	0.63	0.72
210-SER-CB	0.71	0.7
210-SER-HA	0.23	0.23
211-ARG-CA	0.07	0.07
211-ARG-CB	0.81	0.76
211-ARG-HA	0.11	0.11
211-ARG-N	3.07	2.79
213-PRO-C	0.85	0.55
213-PRO-CA	1.52	1.07
213-PRO-CB	0.67	0.32
213-PRO-HA	0.0	0.02
214-SER-C	0.73	0.66
214-SER-CA	0.6	0.89
214-SER-CB	0.07	0.22
214-SER-HN	0.43	0.31
214-SER-HA	0.0	0.02
214-SER-N	3.73	3.31
215-LEU-HN	0.88	0.86
215-LEU-HA	0.08	0.03
215-LEU-N	1.62	2.36
216-PRO-C	0.95	0.97
216-PRO-CA	0.02	0.07
216-PRO-CB	0.25	0.22
216-PRO-HA	0.02	0.01
218-PRO-CA	0.3	0.32
218-PRO-CB	0.18	0.2
218-PRO-HA	0.09	0.11
219-PRO-C	0.05	0.22
219-PRO-CA	0.21	0.35
219-PRO-CB	0.01	0.01
219-PRO-HA	0.04	0.04
220-THR-C	2.15	2.53
220-THR-CA	0.14	0.21
220-THR-CB	0.05	0.11
220-THR-HN	0.1	0.13
220-THR-HA	0.1	0.11
220-THR-N	0.08	0.31
221-ARG-C	0.1	0.16
221-ARG-CA	0.25	0.08
221-ARG-CB	0.14	0.33
221-ARG-HN	0.11	0.14
221-ARG-HA	0.03	0.04
221-ARG-N	0.63	0.8
222-GLU-CA	0.11	0.11
222-GLU-CB	0.23	0.19

	Error [ppm] (native)	Error [ppm] (reweighted)
222-GLU-HN	0.19	0.08
222-GLU-HA	0.18	0.13
222-GLU-N	1.7	1.4
223-PRO-C	0.38	0.36
223-PRO-CA	0.0	0.28
223-PRO-CB	0.15	0.12
223-PRO-HA	0.07	0.07
224-LYS-C	0.4	0.21
224-LYS-CA	1.06	0.03
224-LYS-CB	0.06	0.25
224-LYS-HN	0.04	0.22
224-LYS-HA	0.03	0.07
224-LYS-N	0.02	0.41
225-LYS-C	0.16	0.05
225-LYS-CA	0.76	0.21
225-LYS-CB	0.11	0.14
225-LYS-HN	0.07	0.08
225-LYS-HA	0.08	0.05
225-LYS-N	3.1	1.4
226-VAL-C	0.6	0.38
226-VAL-CA	0.54	0.21
226-VAL-CB	0.12	0.2
226-VAL-HN	0.25	0.09
226-VAL-HA	0.03	0.09
226-VAL-N	2.5	1.11
227-ALA-C	0.32	0.29
227-ALA-CA	0.72	0.04
227-ALA-CB	0.01	0.27
227-ALA-HN	0.33	0.34
227-ALA-HA	0.05	0.02
227-ALA-N	2.51	2.02
228-VAL-C	0.02	0.29
228-VAL-CA	0.28	0.26
228-VAL-CB	0.19	0.02
228-VAL-HN	0.27	0.13
228-VAL-HA	0.07	0.11
228-VAL-N	3.08	2.13
229-VAL-C	0.09	0.12
229-VAL-CA	0.19	0.03
229-VAL-CB	0.27	0.13
229-VAL-HN	0.43	0.15
229-VAL-HA	0.05	0.09
229-VAL-N	3.91	2.0
230-ARG-C	0.28	0.32
230-ARG-CA	0.6	0.71

	Error [ppm] (native)	Error [ppm] (reweighted)
230-ARG-CB	0.27	0.2
230-ARG-HN	0.25	0.19
230-ARG-HA	0.03	0.01
230-ARG-N	2.11	1.38
232-PRO-CA	0.31	0.31
232-PRO-CB	0.24	0.25
232-PRO-HA	0.07	0.06
233-PRO-C	0.26	0.22
233-PRO-CA	0.25	0.24
233-PRO-CB	0.04	0.03
233-PRO-HA	0.03	0.03
234-LYS-C	0.12	0.06
234-LYS-CA	0.34	0.44
234-LYS-CB	0.13	0.15
234-LYS-HN	0.19	0.18
234-LYS-HA	0.01	0.01
234-LYS-N	0.69	0.41
236-PRO-C	0.35	0.36
236-PRO-CA	0.5	0.28
236-PRO-CB	0.14	0.06
236-PRO-HA	0.01	0.02
237-SER-C	0.14	0.25
237-SER-CA	0.14	0.22
237-SER-CB	0.33	0.31
237-SER-HN	0.04	0.08
237-SER-HA	0.01	0.02
237-SER-N	0.58	0.12
238-SER-C	0.5	0.37
238-SER-CA	0.07	0.13
238-SER-CB	0.02	0.15
238-SER-HN	0.08	0.08
238-SER-HA	0.01	0.03
238-SER-N	0.4	0.32
239-ALA-C	0.69	0.7
239-ALA-CA	0.1	0.07
239-ALA-CB	0.03	0.08
239-ALA-HN	0.07	0.08
239-ALA-HA	0.06	0.06
239-ALA-N	0.43	0.07
240-LYS-CA	0.37	0.37
240-LYS-CB	0.17	0.24
240-LYS-HN	0.02	0.03
240-LYS-HA	0.09	0.09
240-LYS-N	3.41	3.2

Figure S24: The error between simulated and experimentally measured chemical shift observables before and after reweighting, using ^3J-couplings and the chemical shifts identified in Stöckelmaier et al. (PCCP 2024, 26, 23856). The reweighting strength was set to $\theta = 0.02$.
